# Identification of Active Components for Sports Supplements: Machine Learning-Driven Classification and Cell-Based Validation

**DOI:** 10.1021/acsomega.3c07395

**Published:** 2024-02-27

**Authors:** Xiaoning Ji, Qiuyun Li, Zhaoping Liu, Weiliang Wu, Chaozheng Zhang, Haixia Sui, Min Chen

**Affiliations:** † State Key Laboratory for Quality Ensurance and Sustainable Use of Dao-di Herbs, National Resource Center for Chinese Materia Medica, China Academy of Chinese Medical Sciences, Beijing 100700, China; ‡ NHC key laboratory of food safety risk assessment, China National Center for Food Safety Risk Assessment, Beijing 100022, China; § NMPA Key Laboratory for Safety Evaluation of Cosmetics, Guangdong Provincial Key Laboratory of Tropical Disease Research, Food Safety and Health Research Center, School of Public Health, Southern Medical University, Guangzhou 510515, China

## Abstract

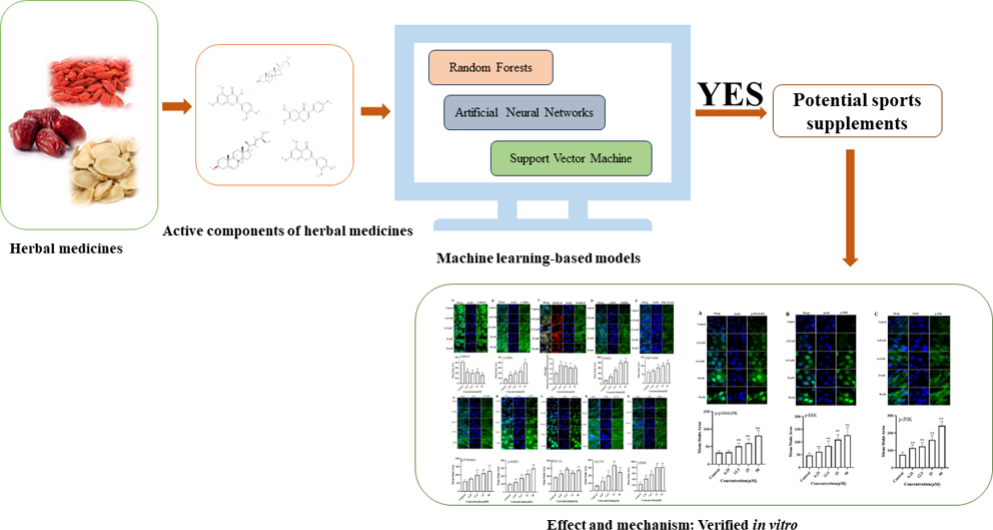

The identification of active components is critical for the development of sports supplements. However, high-throughput screening of active components remains a challenge. This study sought to construct prediction models to screen active components from herbal medicines via machine learning and validate the screening by using cell-based assays. The six constructed models had an accuracy of >0.88. Twelve randomly selected active components from the screening were tested for their active potency on C2C12 cells, and 11 components induced a significant increase in myotube diameters and protein synthesis. The effect and mechanism of luteolin among the 11 active components as potential sports supplements were then investigated by using immunofluorescence staining and high-content imaging analysis. It showed that luteolin increased the skeletal muscle performance via the activation of PGC-1α and MAPK signaling pathways. Thus, high-throughput prediction models can be effectively used to screen active components as sports supplements.

## 1 Introduction

Sports supplements are a class of dietary supplements that are used to improve nutrition and energy and relieve exercise fatigue and injury and are mainly needed for professional exercisers and recreational exercisers.^1−3^ The identification of active components is crucial for the development of sports supplements. Typically, novel sports supplements are investigated by enhancing current compounds, scrutinizing molecular databases, analyzing botanical extracts, and understanding physiological mechanisms, which is time-consuming and expensive. However, the rapid and valid screening of active components remains a considerable challenge. The complex active components in herbal medicines are considered as resources in the development of natural sports supplements that may have favorable efficacy and safety.^4,5^ Most studies have focused on the effect of herbal medicines aimed at enhancing physical performance with insufficient screening of functional components. However, the discovery of natural sports supplements from herbal medicines was serendipitous without extensive screening of the associated bioactivity. Recent advancements in the field of herbal medicine have seen a growing emphasis on the development of machine learning which encompasses a range of applications, including quality control, identification of geographic origins, pharmacodynamic material basis, medicinal properties, and pharmacokinetics and pharmacodynamics.^6,7^ Furthermore, machine learning methods are being utilized to uncover active compounds within herbal medicines, predict their pharmacological activities, and assess potential therapeutic effects.^8,9^ These innovative approaches not only offer new insights into the mechanisms of action of herbal medicines but also provide valuable tools for drug discovery. Therefore, developing prediction models derived via machine learning is essential in screening bioactive components of herbal medicines as potential sports supplements.

Anabolic agents, often referred to as anabolic steroids, are abundant and widely used for doping, which are strictly prohibited by the World Anti-Doping Agency (WADA). Their serious side effects and health risks cannot be ignored and include endocrine disorders, organ toxicity, increased disease risks, drug dependence, reproductive damage, unfair competition, unethical behavior promotion, and high abuse potential.^10^ Despite their reputation, anabolic agents can increase anabolic effects, stimulate growth of skeletal muscle, improve strength, and enhance exercise performance.^11^ This anabolic effect can be demonstrated by the increase in the diameter of myotubes and enhanced synthesis of proteins in C2C12 cells.^12,13^ The chemical structures and biological activities of anabolic agents provide a direct basis for constructing prediction models driven by machine learning. This enables the model to have high accuracy and feasibility and thereby improves the output and precision of research. Machine learning models can preliminarily predict potential active components in herbal medicines, but their practicality and accuracy still require evaluation and validation through supplemental experimental methods.

In this study, we established prediction models using machine learning that used molecular descriptors of anabolic agents. These models were employed to screen for active components of herbal medicines as potential sports supplements. The screened components were randomly validated by the measurement of myotube diameters and protein syntheses in C2C12 cells. The effect and mechanism of active components as potential sports supplements were then investigated by using immunofluorescence and high-content imaging analysis, mainly to assess the capacity of the skeletal muscle related to exercise performance, such as protein synthesis, mitochondrial function, oxidant stress, and glucose uptake.

## 2 Materials and Methods

### 2.1 Chemicals and Reagents

Androstanolone (purity ≥98%) was purchased from Solarbio Science & Technology Ltd. (Beijing, China). Quercetin (purity ≥98%), luteolin (purity ≥98%), kaempferol (purity ≥98%), baicalein (purity ≥98%), calycosin (purity ≥98%), daidzein (purity ≥99%), genistein (purity ≥98%), (+)-catechin (purity ≥98%), naringenin (purity ≥98%), myricetin (purity ≥97%), and prunetin (purity ≥99%) were all purchased from Mackin Regent (Shanghai, China). Tectorigenin (purity ≥98%) was purchased from Shanghai Yuanye Bio-Technology Co., Ltd. (Shanghai, China). Dulbecco’s modified Eagle’s medium (DMEM), fetal bovine serum (FBS), horse serum, phosphate-buffered saline (PBS, pH 7.4), and penicillin-streptomycin were provided by Gibco. A Cell Counting Kit-8 (CCK-8) was obtained from DOJINDO (Japan).

### 2.2 Machine Learning Model Establishment

#### 2.2.1 Data Collection and Curation

Anabolic agents identified as prohibited doping substances were obtained from the WADA Prohibited List (https://www.wada ama.org/sites/default/files/resources/files/2022list_final_en.pdf), which defined them as “positive” chemicals (Table S1). Anabolic agents are synthetic substances that have a chemical structure similar to that of natural testosterone, which acts as an endogenous agonist for the androgen receptor (AR). Therefore, compounds with a similar structure to testosterone but lacking the ability to activate AR were chosen as “negative” candidates to construct the model (Table S1). Chemical activity was derived from National Center for Biotechnology Information, which provides a large suite of online resources for biological information and data, including the GenBank nucleic acid sequence database and the PubMed database of citations and abstracts published in life science journals.^14^

#### 2.2.2 Feature Extraction

Molecular descriptors were generated from the SMILES codes using RDKit package (https://www.rdkit.org) and PyChem (https://academic.oup.com/bioinformatics/article/29/8/1092/233093?login=true). The RDKit descriptor is a general term used in machining learning that encodes chemical structures in their two-dimensional space and includes 208 molecular descriptors. Pychem is an open-source python package for cheminformatics that calculates commonly used structural and physicochemical features and includes 632 fingerprint descriptors, which were used as parameters for model building in this study.

#### 2.2.3 Model Building

To construct highly effective classifiers for identifying promising sports supplements, six prediction models were built using three machine learning algorithms: support vector machine, random forests, and artificial neural networks, as well as two molecular descriptors. All algorithms were achieved in Python 3.9 (https://www.python.org/) with scikit-learn package (https://scikit-learn.org/stable/).

#### 2.2.4 Model Evaluation

Metrics, including accuracy, precision, recall, and F1 score, were used for evaluating the model performance. Parameters were calculated using the following formulas with the number of true positives (TP), true negatives (TN), false positives (FP), and false negatives (FN)
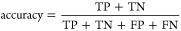
1
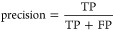
2
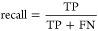
3
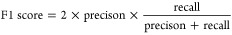
4

In addition, the area under the receiver operating characteristic curve (AUC) was calculated to evaluate the reliability of the classifiers, whose value ranges from 0.5 to 1, where a larger AUC indicates a higher classification performance.

#### 2.2.5 Model Application

Fourteen herbal medicines that are used for both medicine and food and have been related to improving exercise performance in previous study were involved in the current study, including *Radix astragali*, *Panax guinquefolius*, *Codonopsis pilosula*, *Angelica sinensis*, *Fructus lycii*, *Ganoderma lucidum*, *Rhizoma polygonat*, *Poria cocos*, *Pueraria*, *Jujube*, *Liquorice*, *Cistanche*, *Mulberry*, and *Hippophae rhamnoides*.^15−18^ The major chemical active components of these herbal medicines were obtained from the Traditional Chinese Medicine Systems Pharmacology Database and Analysis Platform (TCMSP, https://old.tcmsp-e.com).^19,20^ Finally, 411 chemical active components of the selected herbal medicines were obtained, and molecular descriptors were generated from the SMILES codes using the RDKit package and PyChem as described above. Then, six prediction models were performed for rapid screening of chemical active components that may have a potential anabolic effect. Machine learning-driven models identified active components as “1,” which indicated a high potential anabolic effect, or “0,” which indicated a lower probability of a potential anabolic effect.

### 2.3 *In Vitro* Experiments

#### 2.3.1 Cell Culture and Differentiation

Skeletal muscle cells (C2C12 myoblasts) obtained from Pythonbio (Guangzhou, China) were cultured in growth medium containing DMEM, 10% FBS, and 1% penicillin-streptomycin. At 70–80% confluence, the cells were cultured in DMEM supplemented with 2% horse serum and 1% penicillin-streptomycin for 6 days to induce differentiation into myotubes. The cells were maintained in a humidified 5% CO_2_ at 37 °C. Media were replaced every 2 days.

#### 2.3.2 Cytotoxicity Test

After differentiation, C2C12 myotubes were seeded in 96-well flat-bottom plates at 1 × 10^4^ cells per well and incubated for 24 h. Media were replaced with that containing androstanolone, quercetin, luteolin, kaempferol, baicalein, calycosin, daidzein, genistein, (+)-catechin, naringenin, myricetin, prunetin, or tectorigenin at concentrations from 0.001 to 100 μM, and cultures were incubated for a further 24 h. Following PBS washing, the cytotoxicity of sample treatments was assessed on C2C12 myotubes through the CCK-8 assays. After incubation for 1 h, the absorbance of each well was recorded at 450 nm by using a microplate reader (BioTek Instruments). The experiment was performed in triplicate.

#### 2.3.3 Measurement of Myotube Diameters

Cells were seeded in 6-well plates at 1 × 10^4^ cells/mL (2 mL/well) and were incubated and differentiated as described above. C2C12 myotubes were then treated with each of the sample treatments described above for 24 h. The treatment concentrations of the samples were determined with reference to the toxicity results (Figure S1). Finally, the diameters of the myotubes were measured under a light microscope (Primovert, Zeiss, Germany). Five fields were randomly selected, and 10 myotubes were measured per field using ImageJ (National Institute of Health) as previously described.^21^

#### 2.3.4 Measurement of Protein Synthesis

The method of surface sensing of translation (SUnSET) was used to measure protein synthesis rate.^22^ Puromycin (10 μg/mL) was added into the culture medium and incubated for the last 60 min of treatments. The effect of puromycin incorporation on the total protein expressed was analyzed via Western blotting. C2C12 myotubes were washed twice with cold PBS and lysed using 200 μL of immunoprecipitation assay lysis buffer (Beyotime) that contained 1% PMSF (Panera) and 1% protein phosphatase inhibitor complex (Keygen Biotech). Homogenates were centrifuged at 12,000*g* for 15 min at 4 °C. The total protein content in the sample was quantified by using the Enhanced BCA Protein Assay Kit (Beyotime), according to the manufacturer’s protocol. Samples (50 μg) were electrophoresed on 10% SDS polyacrylamide gels at 120 V for 1.5 h. Proteins were transferred to poly(vinylidene fluoride) (PVDF) membranes and then blocked with 5% (wt/vol) nonfat dry milk in Tris-buffered saline plus 0.1% Tween-20 for 2 h at room temperature. PVDF membranes were then incubated with puromycin antibody (Kerafast) at 4 °C overnight, followed by incubation with antimouse Ig-HRP (Beijing Ray Antibody Biotech) for 1.5 h at room temperature. Protein expression was measured via visualization with enhanced chemiluminescence and ImageJ and was normalized to GAPDH expression. Three independent replicates were performed.

#### 2.3.5 Immunofluorescence Staining and Cell High-Content Imaging Analysis

##### 2.3.5.1 Reactive Oxygen Species (ROS) Measurement

C2C12 myotubes were seeded at 1 × 10^4^ cells per well in a black-wall 96-well plate. The cells were treated with luteolin for 24 h. To induce ROS, the cells were cultured with 100 μM *tert*-butyl hydroperoxide (TBHP) for 30 min. The cells were then stained with CellROX Green reagent (Thermo Fisher Science) for 30 min at 37 °C and protected from light.

##### 2.3.5.2 Lipid Peroxidation Assay

Lipid peroxidation was detected using BODIPY 581/591 C11 (Thermo Fisher Science). Upon oxidation in live cells, the reagent shifts the fluorescence emission peak from 590 to 510 nm (red and green fluorescence, respectively). Following treatment, oxidative stress was induced by exposing C2C12 myotubes to 100 μM TBHP for 2 h. The cells were then washed twice with PBS and incubated in 10 μM BODIPY 581/591 C11 stain for 30 min at 37 °C. The ratio of the signal from the 590 to 510 nm channels was used to quantify lipid peroxidation in cells.

##### 2.3.5.3 Measurement of Mitochondrial Membrane Potential and Mitochondrial Mass

Mitochondrial membrane potential and mitochondrial mass were measured using TMRM reagent (Thermo Fisher Science) and MitoTracker Green FM (Thermo Fisher Science), respectively, according to the manufacturer’s instructions. The cells were washed twice with PBS, then loaded with TMRM staining or MitoTracker at 37 °C for 30 min, and protected from light.

##### 2.3.5.4 Glucose Uptake Assay

C2C12 myotubes were washed with PBS and incubated with glucose-free DMEM (Gibco) containing FBS for 4 h. After being fasted, the cells were treated with 2-NBDG (Thermo Fisher Science) at 400 μM at 37 °C for 30 min, protected from light.

##### 2.3.5.5 Expression of Key Target Proteins

C2C12 cells were fixed with Fixative buffer (eBioscience Fixation:Fixation/Permeabilization, 1:3 ratio) from an eBioscience Foxp3 kit (ThermoFisher Science) at room temperature for 30 min. The cells were then washed twice with permeabilization buffer and incubated with primary antibodies: p-p38 MAPK (Thr180/Tyr182, 1:200, CST), ERK1/2 (Thr202/Tyr204, 1:200, CST), p-JNK (Thr183/Tyr185, 1:200, CST), p-P70S6K1 (Thr389, 1:100, ABclonal), p-4EBP1 (Thr37/Thr46, 1:200, Affinity), GLUT4 (1:200, Abcam), PGC-1α (1:200, Sigma-Aldrich), and HSP60 (1:1000, CST) at room temperature for 30 min. After being washed twice, the cells were probed using antimouse IgG or antirabbit IgG (1:1000, CST) for 1 h at room temperature.

After completion of the previous step, the cells were washed twice with PBS or Cell Staining Buffer (Biolegend). Finally, the cells were counterstained for nuclei with Hoechst 33342 (Solarbio) and analyzed with Image Xpress software (Molecular Device, LLC, San Jose, CA). Each experiment had three biological replicates.^23^

### 2.4 Statical Analysis

Statistical analysis of the data was performed using GraphPad Prism 7.0 (GraphPad Software, Inc., San Diego, CA). Differences were analyzed by one-way analysis of variance followed by Fisher’s least significant difference multiple comparisons. Significant difference was indicated by a *P*-value <0.05.

## 3 Results and Discussion

### 3.1 Constructed Model with High Accuracy Evaluation

**Figure 1:**
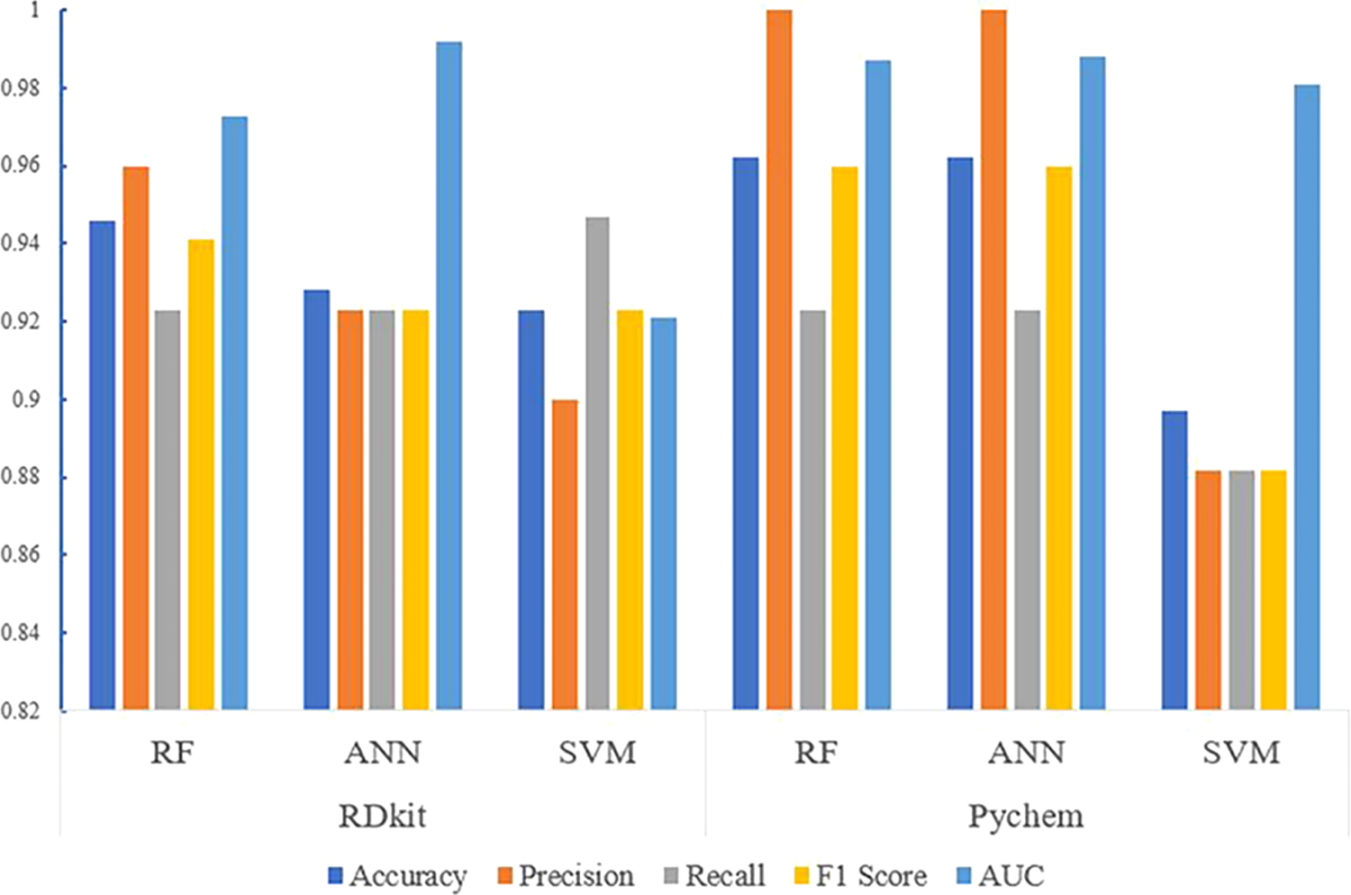
Performance of validation models. RF, Random Forests; ANN, Artificial Neural Networks; SVM, Support Vector Machine; AUC, Area Under the Receiver Operating Characteristic Curve.

The data set analysis is shown in Text S1 and Tables S1–S2. Six machine learning-driven models were developed for rapid and efficient evaluation of active components. The optimal algorithmic hyperparameters of six models were determined using the grid search method (Table S3). The model robustness was evaluated by a 10-fold cross-validation of the training test, and the model generalization ability was evaluated by the test set. Five metrics, including AUC, accuracy, precision, recall, and F1 score, were calculated to evaluate the performance of each model (Figure 1). All values were >0.88.

The number of samples in “positive” database was small, which may impair the performance of machine learning models. Therefore, the Synthetic Minority Oversampling Technique (SMOTE), which analyzed these minority samples, and artificially synthesized new ones to be added to the data set were used to balance the numbers of “positive” and “negative” chemicals. SMOTE has been shown to be effective in mitigating the impact of class imbalance on model performance and has been widely adopted in various machine learning applications, including classification, regression, and anomaly detection, taking into consideration several parameters mainly including the oversampling ratio, neighbor sample selection method, feature space handling, and randomness control.^24,25^ The evaluation indicators of the six machine learning models reconstructed by SMOTE are detailed in Figure S3, which shows that evaluation indicators of the original models were slightly better than those of the reconstructed models.

### 3.2 Application Activity Prediction

A total of 411 active components of 14 kinds of herbal medicines were predicted by the prediction models. In total, 96 chemical active components were identified as “1” by ≥3 prediction models while 43 chemical active components were identified as “1” by ≥4 prediction models; detailed information is shown in Table S4. We adopted a strategy for further result selection whereby the results of these six models were all comprehensively considered rather than considering results from a single model. For example, for the same compound, the more models that predicted this as “1,” the more likely it was to have an anabolic effect and to become a sports supplement.

Several herbal medicines have been shown to have beneficial effects on improving exercise capacity, accelerating recovery, and maintaining health and fitness during intense periods of training.^26^ However, the effective components were still unclear, and barriers remain to their development and application. In this study, we used machine learning to systematically discover natural components that could have potential as sports supplements. After a thorough evaluation, we chose active components that were consistently classified as “1” by at least four prediction models, thus indicating that these were highly promising candidates for sports supplements. Finally, 43 active components were included that mostly had multiple physiological activities and exhibited a high potential for being active components for sports supplements. To validate the screening results, 12 hits were randomly selected for further *in vitro* validation.

### 3.3 Functional Evaluation of Selected Hits

#### 3.3.1 Screening Hits Myotube Diameters

Twelve selected active components were selected for functional validation *in vitro* via evaluating the diameters of differentiated C2C12 myotubes and synthesis of protein in these myotubes.^12^ Androstanolone, a typical anabolic agent, was used as a positive control in our study, and cells cultured with androstanolone formed myotubes whose diameters were significantly larger than those of the control cells (*P* < 0.05; Figure S4). Moreover, exposure to 11 active components increased myotube diameters relative to the control, as shown in Figure S5. The effect was particularly pronounced at higher concentrations. Notably, the inclusion of baicalein did not produce any significant enhancement in myotube diameters versus the control.

#### 3.3.2 Screening Hits Protein Synthesis

We then assessed the protein synthesis in C2C12 myotubes. As demonstrated in Figure S6, treatment with 11 of the active components significantly increased protein synthesis as evidenced by the degree of puromycin incorporation (*P* < 0.05). However, baicalein treatment did not produce a significant increase in protein synthesis as the negative result observed in myotube diameters. Cultured cells treated with androstanolone exhibited a substantial increase in protein synthesis (*P* < 0.05; Figure S7).

The prediction results of the machine learning-driven models and *in vitro* experiments are summarized in Table 1. We observed that 11 of the 12 randomly selected active components of herbal medicines increased the myotube diameter and promoted protein synthesis in C2C12 myotubes, showing good generalization ability of machine learning-driven models. The prediction models constructed by machine learning may improve the efficiency of screening active compounds that can enhance exercise performance and facilitate the further development of active components in herbal medicines.

**Table 1:** 12 Active Components of Herbal Medicines and Their Prediction and Experimental Results^a^

		RDkit			Pychem			*in vitro* validation	
CID		RF	ANN	SVM	RF	ANN	SVM	myotube diameters	protein synthesis
luteolin	5280445	1	1	1	1	1	1	√	√
baicalein	5281605	1	1	0	0	1	1	×	×
quercetin	5280343	1	1	0	0	1	1	√	√
calycosin	5280448	1	1	0	0	1	1	√	√
kaempferol	5280863	1	1	0	0	1	1	√	√
daidzein	5281708	0	1	1	0	1	1	√	√
genistein	5280961	1	1	0	0	1	1	√	√
(+)-catechin	9064	1	1	1	0	1	1	√	√
naringenin	932	1	1	1	1	1	1	√	√
myricetin	5281672	1	1	0	1	1	1	√	√
prunetin	5281804	1	1	0	0	1	1	√	√
tectorigenin	5281811	1	1	0	0	1	1	√	√

a Note: √, Active component of herbal medicines had the effect of increasing diameter or protein synthesis in C2C12; ×, Active component of herbal medicines had no effect of increasing diameter or protein synthesis in C2C12 in the vitro experiment; 1, chemical active component had higher probability of potential of anabolic effect predicted by machine learning-driven model; 0, lower probability of potential of anabolic effect predicted by machine learning-driven model.

We confirmed that 11 active components of 12 hits had an efficacious anabolic effect. The identified active components were natural components of herbal medicines that efficaciously stimulated muscle growth and might play important roles in sports performance and overall health. Quercetin and (+)-catechin are polyphenolic flavonoids and powerful antioxidants and are the main components found in medicinal herbs that have beneficial effects including anti-inflammatory, antiallergy, and anticancer activities and supporting liver and heart health.^27−29^ Myricetin treatment is associated with an increase in the proportion of slow-twitch myofibers, and daidzein alleviates cisplatin-induced muscle hypertrophy.^30,31^ Specifically, luteolin, identified as “1” by six prediction models, has been shown to have beneficial effects on skeletal muscle and improved exercise performance.^32^ However, the actions and molecular mechanisms of luteolin on the adaptive capacity of skeletal muscle as novel sports supplements have not yet been explored. To verify the practicality of our models, we conducted an analysis of the effect and molecular mechanisms of luteolin used as a novel sports supplements.

### 3.4 Function and Mechanism of Luteolin as a Sport Supplement

#### 3.4.1 Decreased Oxidative Stress

**Figure 2:**
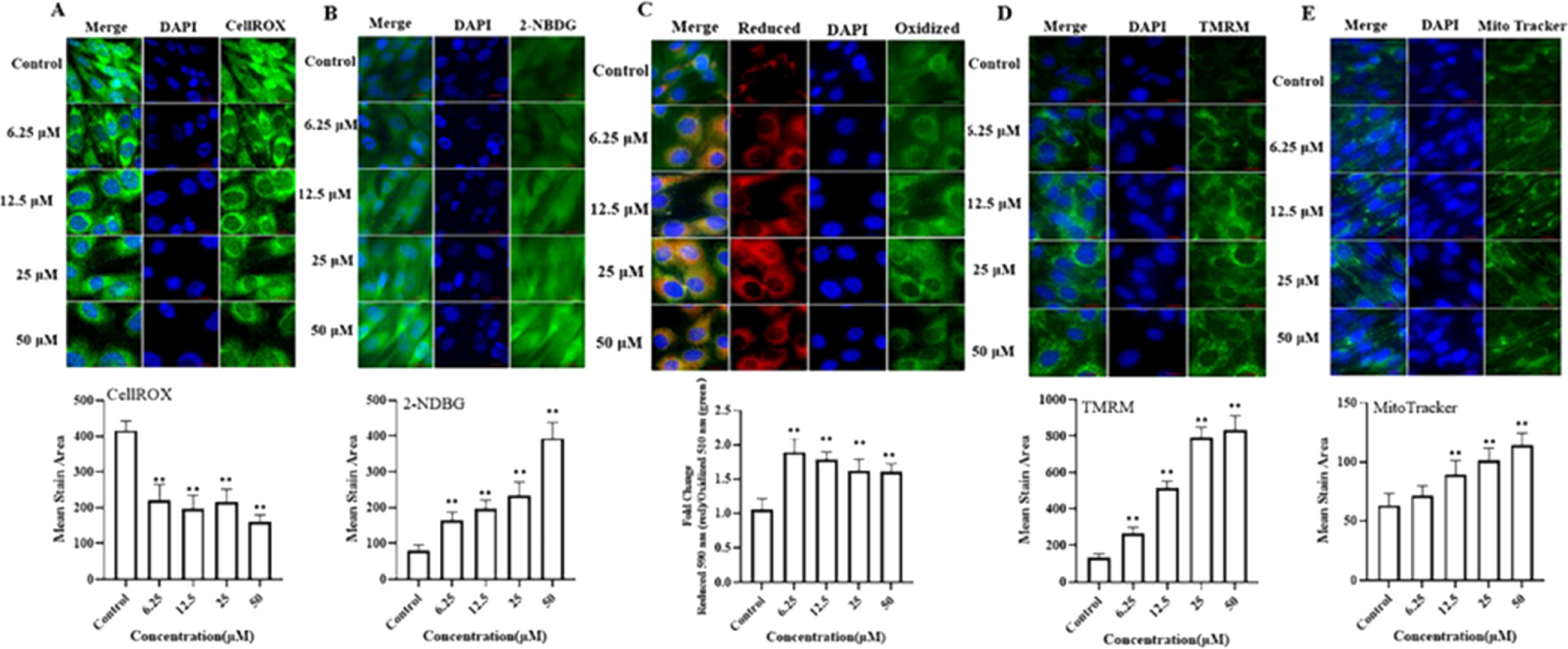
Effect of luteolin on glucose uptake, ROS generation, lipid peroxidation, and mitochondria function in C2C12 myotubes. The mean stained area of CellROX (A), BODIPY 581/591 C11 (B), 2-NBDG (C), TMRM (D), and MitoTracker (E) probes was detected with a high-content imaging analysis system. Left: representative images, bar size: 50 μm. *, *P* < 0.05; **, *P* < 0.01.

To determine the specific role of luteolin in antioxidant activity, CellROX staining was used to quantify cellular ROS. Preincubation with luteolin at 6.25, 12.5, 25, and 50 μM significantly reduced the oxidative stress-induced increase in ROS concentration (Figure 2A).

Lipid peroxidation was identified using BODIPY 581/591 C11 dye. Oxidation of cells resulted in a shift of the fluorescence emission peak from approximately 590 to approximately 510 nm. A lower 590:510 ratio indicated a higher degree of oxidation, whereas a higher ratio indicated a lower degree of oxidation. As illustrated in Figure 2B, reduced lipid peroxidation in cells pretreatment with luteolin was demonstrated by the increased 590:510 ratio in the luteolin-treated group.

Excessive ROS during exercise can affect the normal function of cells and reduce muscle contraction and endurance.^33^ Luteolin exhibited potent antioxidant properties, as evidenced by the reduction of levels of ROS and lipid peroxidation. High levels of lipid peroxidation can induce a range of damaging effects such as cell membrane dysfunction, DNA damage, and inflammation. Reducing oxidative stress may potentially decrease exercise-induced harm and inflammation, enabling a quicker recovery and enhancing performance in both training and competition.^34^

#### 3.4.2 Increased Glucose Uptake

Glucose uptake by C2C12 myotubes was explored using 2-NBDG, a fluorescently labeled glucose analogue, to assess the effect of luteolin treatment on glucose uptake. The results indicated glucose uptake in the luteolin-treated group was significantly higher than that in the control group (Figure 2C). Glucose serves as a primary material for generating ATP, which is critical in supplying energy to varying exercise intensities, thereby increasing the exercise time and intensity. Furthermore, glucose is an essential source of energy for neuromuscular function and cognitive activity. Insufficient intake of glucose adversely affects the work efficiency of neuromuscular and cognitive function, causing a decline in exercise coordination.^35^ In addition, the enhanced uptake of glucose may be advantageous in mitigating protein and muscle damage.

#### 3.4.3 Enhanced Mitochondrial Function

Mitochondria are the main sites for producing ATP, and a strong mitochondrial function can directly affect exercise capacity and physical fitness.^36^ Mitochondrial membrane potential and mitochondrial mass were mainly used as indicators of key parameters of mitochondrial health and function. The TMRM and MitoTracker stained areas were significantly larger in size in cells treated with luteolin (6.25–50 μM for 24 h) than stained areas in control cells (Figure 2D–E). The stronger mitochondrial function provides an energy base for exercise and directly affects the aerobic exercise capacity and endurance.^37^ A stronger mitochondrial function is also conducive to muscle gain and strength increase to enhance exercise capacities, such as power, speed, and strength.^36^

#### 3.4.4 Effects of Luteolin on MAPK Signaling Pathway

**Figure 3:**
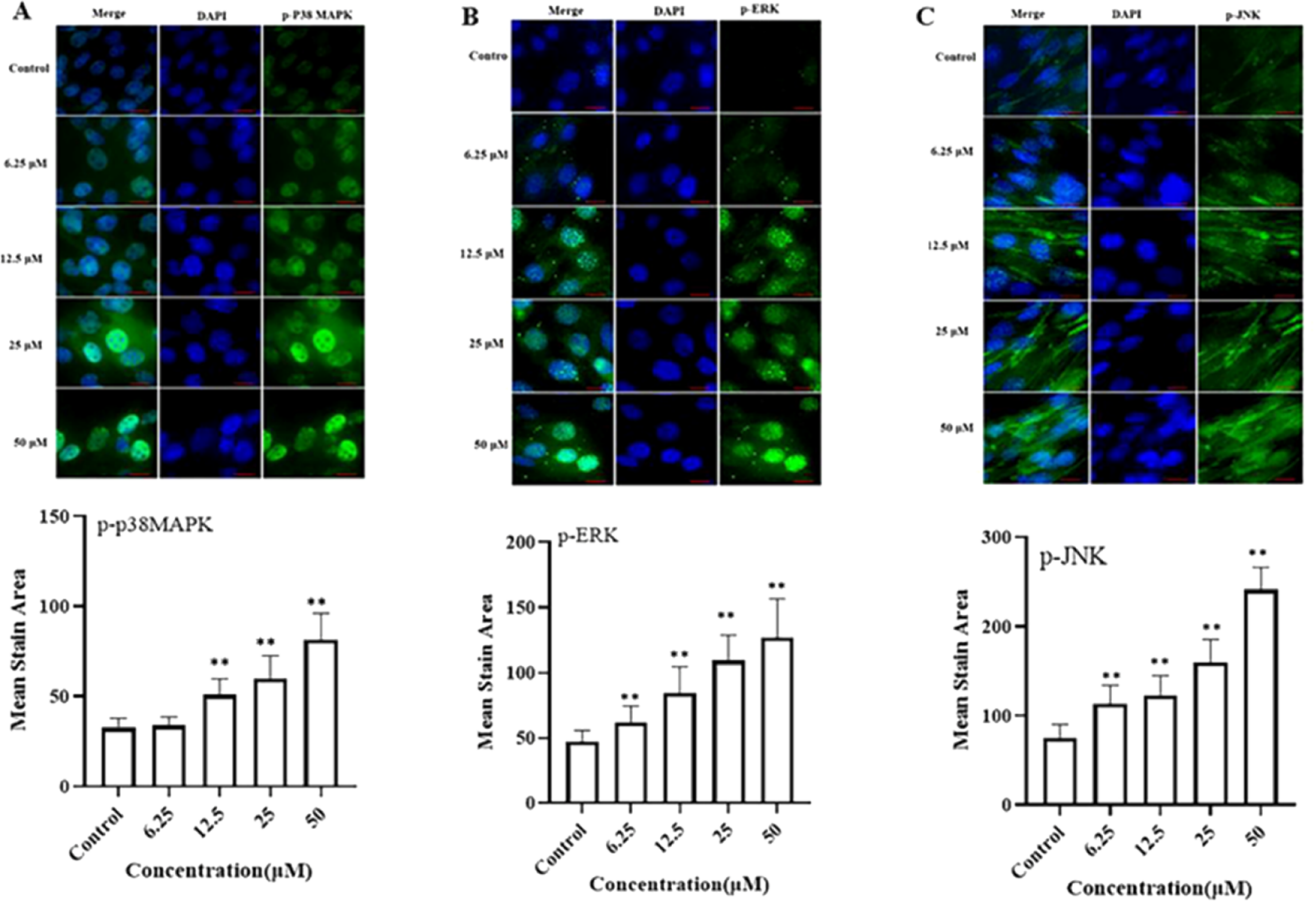
Protein expression of markers of the MAPK signaling pathway. Protein expression of p-p38 MAPK (A), p-ERK(B), and p-JNK (C) was detected with a high-content imaging analysis system. Top: representative images, bar size: 50 μm. *, *P* < 0.05; **, *P* < 0.01.

The MAPK signaling pathway is associated with multiple biological functions, especially in energy metabolism.^35^ To characterize the underlying mechanisms for the effect of luteolin on the MAPK signaling pathway, we evaluated the levels of phosphorylation of marker proteins in the MAPK signaling pathway, including those of p38 MAPK, ERK, and JNK. Luteolin enhanced the phosphorylation of p38 MAPK, ERK, and JNK (Figure 3A–C), which implied that luteolin could activate the MAPK signaling pathway to exert its physiological functions. Consistent with our results, previous studies have shown that the MAPK signaling pathway is involved in regulating glucose uptake, protein synthesis, and mitochondrial biogenesis in skeletal muscle.^38,39^

#### 3.4.5 Activated P70S6K1, 4EBP1, PGC-1a, HSP60, and GLUT4

**Figure 4:**
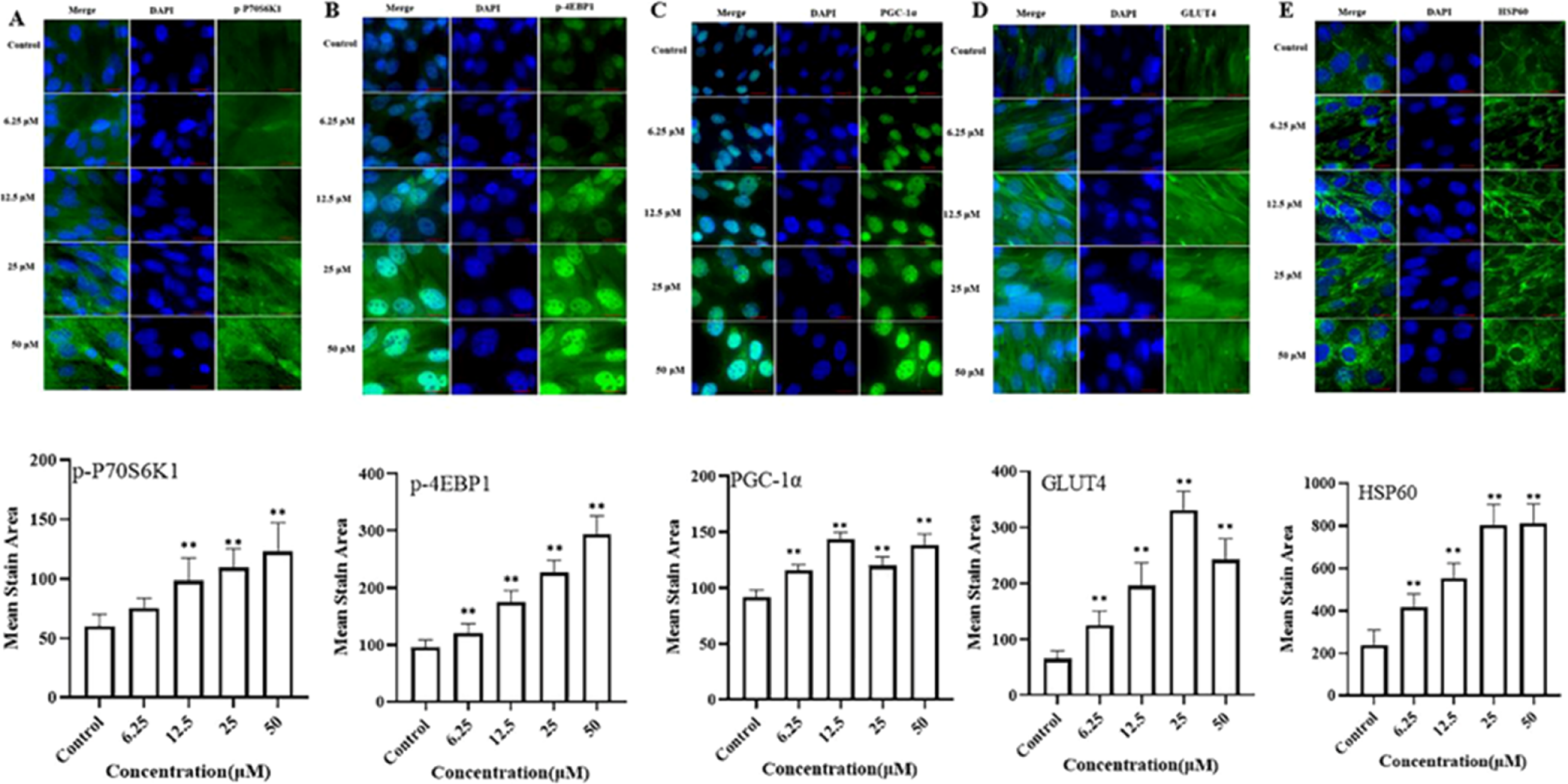
Protein expression of key downstream targeted proteins in C2C12 myotubes. Protein expression of p-P70S6K1 (A), p-4EBP1 (B), PGC-1α (C), GLUT4 (D), and HSP60 (E) was detected with a high-content imaging analysis system. Top: representative images, bar size: 50 μm. *, *P* < 0.05; **, *P* < 0.01.

To investigate the underlying molecular mechanism of the observed luteolin effects, we evaluated the protein expression upstream of the MAPK signaling pathway and conducted a direct analysis of the downstream targets specifically associated with function. Luteolin treatment increased phosphorylation of P70S6K1 and 4EBP1 (Figure 4A,B). The enhanced phosphorylation of these proteins is strongly correlated with protein synthesis as indicated by a significant increase in protein synthesis.^40^ Furthermore, the phosphorylation of these sites by ERK and p38 MAPK is suggested to promote mTORC1 activity and signaling to downstream substrates, such as 4EBP1 and P70S6K1.^41,42^ PGC-1α has been highlighted as a transcriptional coactivator that regulates energy metabolism via mitochondrial biogenesis, oxidative metabolism, and muscle growth.^43,44^ Hence, our investigation entailed an analysis of the expression of the PGC-1α protein. Luteolin inculcation with C2C12 myotubes considerably increased the expression of PGC-1α, HSP60, and GLUT4 (Figure 4C–E). Elevated glucose uptake has been correlated with the stimulation of PGC-1α and upregulation of GLUT4 expression.^45,46^ Additionally, PGC-1α is a core factor of mitochondrial function and has been shown to upregulate the expression of HSP60, which assists in maintaining the stability and appropriateness of mitochondrial proteins, thus enhancing the overall functionality of mitochondria;^47,48^ PGC-1α can also increase protein synthesis.^49^ Phosphorylation of MAPK signaling pathway may also upregulate the expression of PGC-1α.^50^ Thus, luteolin may activate the expression of PGC-1α through MAPK signaling pathways, along with decreasing oxidative stress and increasing protein synthesis, mitochondrial function, and glucose uptake.

## 4 Conclusions

Six prediction models constructed by machine learning were successfully used to screen potential sports supplements from 411 active components identified from 14 types of herbal medicines. In total, 43 chemically active components were consistently classified as active components by at least four prediction models. Twelve active components were randomly selected for further *in vitro* validation, of which 11 active components increased myotube diameter and protein synthesis, showing their high potential as sports supplements. Further investigation of the mechanism of action for luteolin among the 11 active components suggests that luteolin may increase energy metabolism and protein synthesis and decrease oxidative stress to enhance exercise capacity via the activation of expression of PGC-1α and following the enhanced expression of P70S6K1, 4EBP1, GLUT4, and HSP60 through MAPK signaling pathways.

However, some limitations of this study should be considered. This study primarily screened the active components of 14 herbal medicines; the inclusion of a wider range of herbal medicines can be considered in future research. Additionally, it should be noted that this study utilized only the commonly used classic database, TCSMP, to obtain the main active components of herbal medicines, which has certain limitations, especially in potentially overlooking the metabolites of the active components. In subsequent research, the integration of other databases, literature, and detection methods can be employed to improve this aspect of the data. Considering the current limited data set, this screening method could be improved with more mechanism-based modeling and future algorithms despite our attempt to expand the data set by using an up-sampling method. Nevertheless, in conjunction with our experimental findings, our screening method demonstrates a relatively high accuracy toward identifying active components from herbal medicines for sports supplements. Further extensive research is necessary to ascertain the active compounds of herbal medicines that we have evaluated for safety and dose in animals and humans, thus enabling the development of a safer and more efficacious sports supplement.

## Data Availability

Data will be made available upon request.

## Supporting Information Available

Data set analysis (Text S1); data set for modeling (Table S1); number of chemicals in the training and test (Table S2); optimal hyperparameters of three algorithms in six models (Table S3); prediction results of active components of herbal medicines driven by machine learning models (Table S4); CCK-8 assay of cell viability (Figure S1); chemicals spatial distribution (Figure S2); performance of validation for the models (Figure S3); effects of androstanoloneon on the diameter of C2C12 myotubes (Figure S4); effects of androstanoloneon on protein synthesis of C2C12 myotubes (Figure S5); effects of active components on the diameter of C2C12 myotubes (Figure S6); and effects of active components on the protein synthesis of C2C12 (Figure S7) (PDF)
