# Supplementary material for: Identification of Active Components for Sports Supplements: Machine Learning-Driven Classification and Cell-Based Validation
Source: ACS Omega. 2024 Feb 27;9(10):11347–55. doi: 10.1021/acsomega.3c07395 (PMC10938306; doi:10.1021/acsomega.3c07395)
Supplement: Supplementary file 1 — ao3c07395_si_001.pdf [file ao3c07395_si_001.pdf]

Identification of active components for sports supplements: Machine learning-driven classification and cell-based validation

Xiaoning Ji [a, b], Qiuyun Li [c], Zhaoping Liu [b], Weiliang Wu [c], Chaozheng Zhang [b], Haixia Sui [b, *], Min Chen [a, *]

[a] State Key Laboratory for Quality Ensurance and Sustainable Use of Dao-di Herbs, National Resource Center for Chinese Materia Medica, China Academy of Chinese Medical Sciences, Beijing 100700, China

[b] NHC key laboratory of food safety risk assessment, China National Center for Food Safety Risk Assessment, Beijing 100022, China

[c] NMPA Key Laboratory for Safety Evaluation of Cosmetics, Guangdong Provincial Key Laboratory of Tropical Disease Research, Food Safety and Health Research Center, School of Public Health, Southern Medical University, Guangzhou 510515, China

[*] Corresponding authors. E-mail address: suihaixia@cfsa.net.cn (Haixia Sui), cm315keke@163.com (Min Chen).

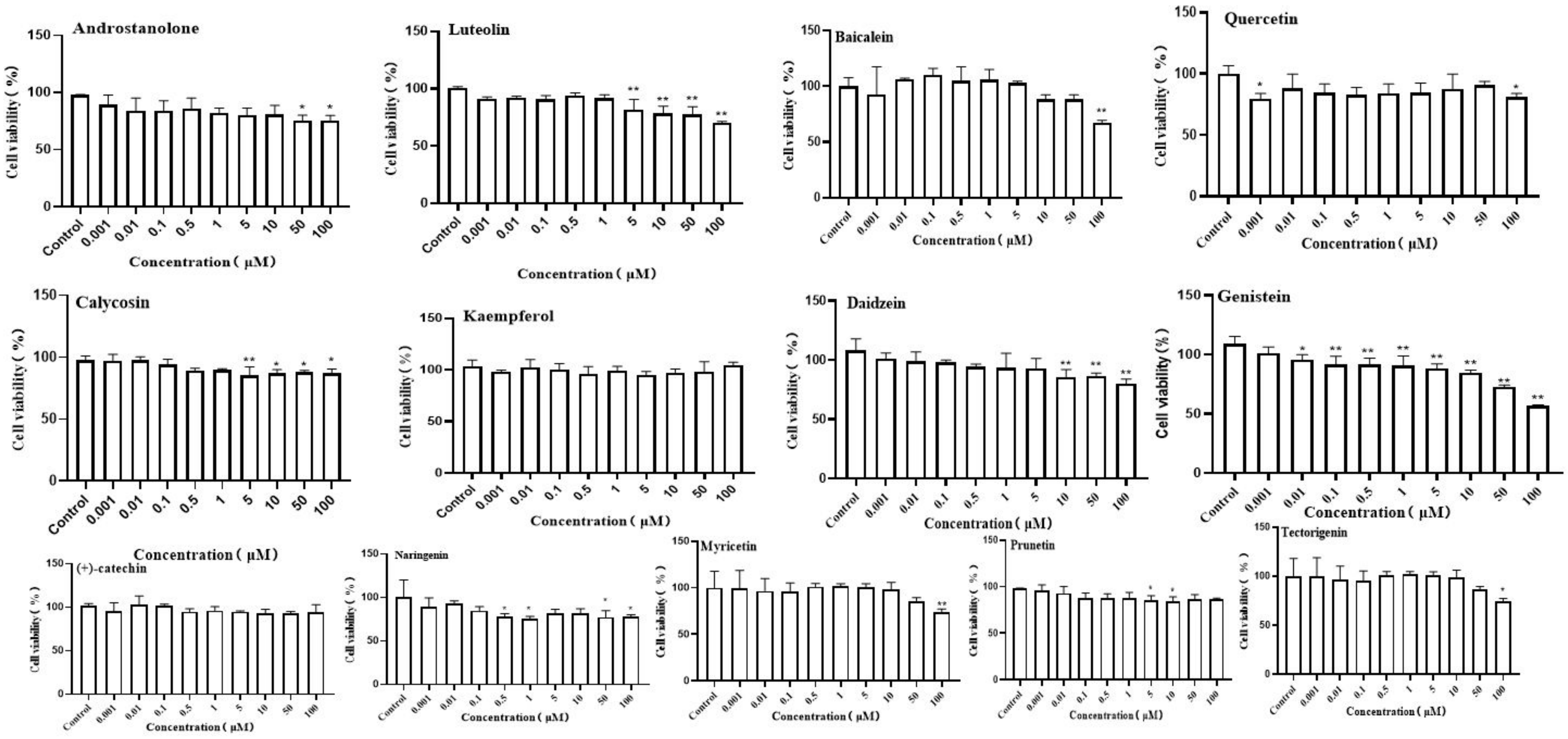

Figure S1. CCK-8 assay of cell viability. *, *P*<0.05; **, *P*<0.01.

Text S1. Data set analysis

72 Anabolic Agents according to the Prohibition List promulgated by WADA were collected as the "positive", and 113 chemicals as the "negative" from PubChem (showed in **Table S1**). After data splitting, the training set and test set contain129 and 56 chemicals, respectively. The "positive" and "negative" chemicals in each date sets were showed in **Table S2.** In order to develop robust prediction models, the chemical space distribution was used to explore the chemical diversity for two data sets in this study. The chemical space distribution defined by three principal components performed by PCA (Principal Component Analysis), which were illustrated in **Figure S2**. The chemical space of the test set was basically contained by the training set, indicating the appropriate division which was beneficial for model predictio[1].

Reference

1. Zhao P, Peng Y, Xu X, et al. In silico prediction of mitochondrial toxicity of chemicals using machine learning methods. *J Appl Toxicol.* Oct 2021;41(10):1518-1526.

Table S1. Data set for modeling

Class	SMILES
0	C[C@]12CC[C@H]3[C@H]([C@@H]1CCC2=O)CC=C4[C@@]3(CC[C@@H](C4)O)C
0	CC(=O)[C@H]1CC[C@@H]2[C@@]1(CC[C@H]3[C@H]2CC=C4[C@@]3(CC[C@@H](C4)O)C)C
0	C[C@H](CCCC(C)C)[C@H]1CC(=O)C2=C3CC[C@H]4C[C@H](CC[C@@]4([C@H]3CC[C@]12C)C)O
0	CC(=O)[C@H]1[C@@H](C[C@@H]2[C@@]1(CC[C@H]3[C@H]2CC=C4[C@@]3(CC[C@@H](C4)O)C)C)C#N
0	C[C@H](CCCC(C)(C)O)[C@H]1CC[C@@H]2[C@@]1(CC[C@H]3[C@H]2CC=C4[C@@]3(CC[C@@H](C4)O)C)C
0	C/C=C(/CC[C@@H](C)[C@H]1CC[C@@H]2[C@@]1(CC[C@H]3C2=CC[C@@H]4[C@@]3(CC[C@@H]([C@H]4C)O)C)C)\C(C)C
0	C[C@]12CC[C@H]3[C@H]([C@@H]1CC[C@]2(CC=C)O)CCC4=CCCC[C@H]34
0	CC(=O)[C@H]1CC[C@@H]2[C@@]1(CC[C@H]3[C@H]2CC[C@@H]4[C@@]3(CC[C@@](C4)(C)O)C)C
0	C[C@H](CCCC(C)C)[C@H]1CC[C@@H]2[C@@]1(CC[C@H]3[C@H]2CC=C4[C@@]3(CC[C@@H](C4)O)C)C
0	C[C@H]\1CC[C@@H](C/C1=C\C=C\2/CCC[C@]3([C@H]2CC[C@@H]3[C@H](C)/C=C/[C@H](C)C(C)C)C)O
0	C[C@H](CCCC(C)(C)O)[C@H]1CC[C@@H]\2[C@@]1(CCC/C2=C\C=C/3\C[C@H](CCC3=C)O)C
0	CC[C@H](CC[C@@H](C)[C@H]1CC[C@@H]2[C@@]1(CC[C@H]3[C@H]2CC=C4[C@@]3(CC[C@@H](C4)O)C)C)C(C)C
0	CC(=O)[C@H]1CC[C@@H]2[C@@]1(CC(=O)[C@H]3[C@H]2CC[C@@H]4[C@@]3(CC[C@H](C4)O)C)C
0	C[C@H](/C=C/[C@H](C)C(C)C)[C@H]1CC[C@@H]\2[C@@]1(CCC/C2=C\C=C/3\C[C@H](CCC3=C)O)C
0	C/C=C\1/C(=O)C[C@@H]2[C@@]1(CC[C@H]3[C@H]2CCC4=CC(=O)CC[C@]34C)C
0	C/C=C/1\C(=O)C[C@@H]2[C@@]1(CC[C@H]3[C@H]2CCC4=CC(=O)CC[C@]34C)C
0	CC([C@H]1CC[C@@H]2[C@@]1(CC[C@H]3[C@H]2CC[C@H]4[C@@]3(CC[C@@H](C4)O)C)C)O
0	C[C@@H]1C[C@H]2[C@@H]3CCC4=CC(=O)C=C[C@@]4([C@H]3[C@H](C[C@@]2([C@@]1(C)C(=O)OC)C)O)C
0	C[C@]12CC[C@](C[C@H]1C3=CC(=O)[C@@H]4[C@]5(CC[C@@H](C([C@@H]5CC[C@]4([C@@]3(CC2)C)C)(C)C)O)C)(C)C(=O)O
0	C[C@@]12CCC[C@H]1[C@@H]3CC[C@H]4C[C@H](CC[C@@]4([C@H]3CC2)C)O
0	C[C@H](CCCC(C)C)[C@H]1CC[C@@H]\2[C@@]1(CCC/C2=C\C=C/3\C[C@H](CCC3=C)O)C
0	C[C@H](/C=C/[C@H](C)C(C)(C)O)[C@H]1CC[C@@H]\2[C@@]1(CCC/C2=C\C=C3C[C@H](C[C@@H](C3)O)O)C

0 C[C@H](/C=C/[C@H](C)C(C)C)[C@H]1CC[C@@H]\2[C@@]1(CCC/C2=C\C=C/3\C[C@H](C[C@@H](C3=C)O)O)C
0 CC(=O)[C@]1(CC[C@@H]2[C@@]1(CC[C@H]3[C@H]2C=C(C4=CC(=O)[C@@H]5C[C@@H]5[C@]34C)Cl)C)O
0 CC(=O)[C@H]1CC[C@@H]2[C@@]1(CC[C@H]3[C@H]2CC=C4[C@@]3(CC[C@@H](C4)OC(=O)C)C)C
0 C[C@H](CCC(=O)O)[C@H]1CC[C@@H]2[C@@]1(CC[C@H]3[C@H]2CC[C@H]4[C@@]3(CC[C@H](C4)O)C)C
0 CC(=O)O[C@H]1CC[C@@]2([C@H]3CC[C@]4([C@H]([C@@H]3CC=C2C1)CCC4=O)C)C
0 C[C@H](CCCC(C)(C)O)[C@H]1CC[C@@H]\2[C@@]1(CCC/C2=C\C=C/3\C[C@H](C[C@@H](C3=C)O)O)C
0 C[C@H](CC[C@H](C(C)(C)O)O)[C@H]1CC[C@@H]\2[C@@]1(CCC/C2=C\C=C/3\C[C@H](CCC3=C)O)C
0 C[C@H](CCCC(C)C)[C@H]1CC[C@@H]2[C@@]1(CC[C@H]3[C@H]2CC[C@H]4[C@@]3(CC[C@H](C4)O)C)C
0 C[C@@H]1CC(=O)C=C2[C@]1(C[C@@H](CC2)C(=C)C)C
0 C[C@H](CCC(=O)O)[C@H]1CC[C@@H]2[C@@]1(CC[C@H]3[C@H]2[C@@H](C[C@H]4[C@@]3(CC[C@H](C4)O)C)O)C
0 C[C@@H]1CC[C@H](C2=C(C[C@H]([C@H]12)O)C)/C=C(\C)/C(=O)O
0 C[C@]12CC[C@H]3[C@H]([C@@H]1CCC2=O)CCC4=C3C=CC(=C4)O
0 CC(C)C1=CC2=CC[C@@H]3[C@@]([C@H]2CC1)(CCC[C@@]3(C)C(=O)O)C
0 C[C@]12CC[C@H]3[C@H]([C@@H]1CC[C@]2(C#C)O)CCC4=C3C=CC(=C4)OC
0 C[C@H](CCC(=O)O)[C@H]1CC[C@@H]2[C@@]1(C(=O)C[C@H]3[C@H]2C(=O)C[C@H]4[C@@]3(CCC(=O)C4)C)C
0 CC(C)(CCCC1=CCC(CC1)C=O)O
0 C[C@]12CC[C@H](C1(C)C)C[C@H]2O
0 C[C@@]12CC[C@@H](C1(C)C)C[C@H]2O
0 C[C@]12CC[C@H]3[C@H]([C@@H]1CC[C@]2(C#C)O)CCC4=C3C=CC(=C4)OC5CCCC5
0 CC1=C[C@@H]2[C@H](CC[C@]3([C@H]2CC(=C)[C@@]3(C(=O)C)OC(=O)C)C)[C@@]4(C1=CC(=O)CC4)C
0 C[C@@H]1CC[C@H]([C@H](C1)O)C(=C)C
0 CC(=C)C1CCC(CC1)(C)O
0 C[C@]12CC[C@H]3[C@H]([C@@H]1CC=C2)CC[C@@H]4[C@@]3(CCC(=O)C4)C
0 CC1C2CC(C1(C)C)CC2C3CCCC(C3)O
0 C[C@H]1[C@@H]2CC[C@]3([C@H]([C@]2(CC[C@H]1O)C)[C@@H](C[C@@H]\4[C@@]3(C[C@@H](/C4=C(/CCC=C(C)C)\C(=O)[O-])OC(=O)C)C)O)C.[Na+]

0 CC1C2C(=O)C3(CCC2(C3CC1=O)C)C(C)C(=O)O

0 CC#CCC(C)[C@@H](/C=C/C1[C@@H](C[C@H]2[C@@H]1C/C(=C/CCCC(=O)O)/C2)O)O

0 C[C@]12CC[C@H]3[C@H]([C@@H]1CCC(=O)O2)CCC4=CC(=O)C=C[C@]34C

0 CCCCCC(=O)O[C@@]1(CC[C@@H]2[C@@]1(CC[C@H]3[C@H]2CCC4=CC(=O)CC[C@]34C)C)C(=O)C

0 C[C@@H]([C@H]1CC[C@@H]\2[C@@]1(CCC/C2=C\C=C/3\C[C@H](C[C@@H](C3=C)O)O)C)OCCC(C)(C)O

0 CC1C(C2=C(C1(C)C)C(=O)CCC2)(C)C

0 CC1=CCC(CC1)(C(C)C)O

0 CC(=C)C(=O)O[C@@H]1C[C@H]2CC[C@@]1(C2(C)C)C

0 C[C@]12CC[C@H]3[C@H]([C@@H]1C[C@H]([C@@H]2O)O)CCC4=C3C=CC(=C4)O

0 CC(=O)O[C@H]1CC[C@@]2([C@H]3CC[C@]4([C@H]([C@@H]3CC=C2C1)CC=C4C5=CN=CC=C5)C)C

0 C[C@H](CCC(=O)[O-])[C@H]1CC[C@@H]2[C@@]1([C@H](C[C@H]3[C@H]2[C@@H](C[C@H]4[C@@]3(CC[C@H](C4)O)C)O)O)C.[Na+]

0 C[C@H](CCC(=O)[O-])[C@H]1CC[C@@H]2[C@@]1([C@H](C[C@H]3[C@H]2CC[C@H]4[C@@]3(CC[C@H](C4)O)C)O)C.[Na+]

0 CC(=O)OCC(=O)[C@]1([C@@H](C[C@@H]2[C@@]1(C[C@@H]([C@]3([C@H]2CCC4=CC(=O)C=C[C@@]43C)F)O)C)OC(=O)C)O

0 C[C@@H]1CC[C@@H]2[C@]13CC[C@@]([C@H](C3)C2(C)C)(C)O

0 C[C@@H]1CC[C@@]2([C@H]([C@H]3[C@@H](O2)C[C@@H]4[C@@]3(C(=O)C[C@H]5[C@H]4CC[C@@H]6[C@@]5(CC[C@@H](C6)O)C)C)C)OC1

0 CC1CC2=C(CC1(C)C(=O)C)C(CCC2)(C)C

0 C[C@@]12CC[C@H]([C@@]([C@H]1CCC(=C)[C@H]2C/C=C/3\[C@@H](COC3=O)O)(C)CO)O

0 C[C@H]1[C@@H]2CC[C@]3(C=CC(=O)C(=C3[C@H]2OC1=O)C)C

0 CC[C@H](C)C(=O)O[C@H]1CCC=C2[C@H]1[C@H]([C@H](C=C2)C)CC[C@@H]3C[C@H](CC(=O)O3)O

0 CC1=CC[C@H](CC1)[C@](C)(CCC=C(C)C)O

0 CC1=CC[C@@H](CC1)[C@@](C)(CCC=C(C)C)O

0 CC1(CCC(CC1)C(C)(C)O)O

0 C[C@]12C[C@@H]([C@H]3[C@H]([C@@H]1C[C@@H]4[C@]2(OC(O4)(C)C)C(=O)CO)CCC5=CC(=O)C=C[C@]35C)O
0 C[C@@]12[C@H](C=C[C@@]3([C@@H]1[C@@H]([C@]45[C@H]3CC[C@](C4)(C(=C)C5)O)C(=O)O)OC2=O)O
0 C[C@]12CC[C@](C[C@H]1C3=CC(=O)[C@@H]4[C@]5(CC[C@@H](C([C@@H]5CC[C@]4([C@@]3(CC2)C)C)(C)C)O[C@@H]6[C@@H]([C@H]([C@@H]([C@H](O6)C(=O)O)O)O)O[C@H]7[C@@H]([C@H]([C@@H]([C@H](O7)C(=O)O)O)O)O)C)(C)C(=O)O
0 CC(C)(C1CCC(CC1)O)C2CCC(CC2)O
0 C[C@@H]1CC[C@H](C2=C(CC[C@H]12)C)/C=C(\C)/C(=O)O
0 C[C@@]12CC[C@@H](C1)C([C@@H]2O)(C)C
0 CC1(CCC(=O)[C@@H]2C13CCC(C3)C2(C)C)C
0 CC1(C2CCC(C2)(C1O)C)C
0 CC(=O)[C@]1(CC[C@@H]2[C@@]1(CC[C@H]3[C@H]2C=C(C4=CC(=O)[C@@H]5C[C@@H]5[C@]34C)Cl)C)OC(=O)C
0 CC1=C(C(=O)C(=C(C1=O)C)C(CCCCCC(=O)O)C2=CC=CC=C2)C
0 CC[C@H](C)C(=O)O[C@H]1C[C@@H](C=C2[C@H]1[C@H]([C@H](C=C2)C)CC[C@H](C[C@H](CC(=O)[O-])O)O)O.[Na+]
0 CC(=O)O[C@@H]1CC2CCC1(C2(C)C)C
0 CC(=O)O[C@@H]1C[C@H]2CC[C@@]1(C2(C)C)C
0 C[C@]12CC[C@](C[C@H]1C3=CC(=O)[C@@H]4[C@]5(CC[C@@H](C([C@@H]5CC[C@]4([C@@]3(CC2)C)C)(C)C)OC(=O)CCC(=O)[O-])C)(C)C(=O)[O-].[Na+].[Na+]
0 C[C@H]1C[C@@H]2[C@H]([C@H]([C@]3(O2)CC[C@H]4[C@@H]5CC=C6C[C@H](CC[C@@]6([C@H]5CC4=C3C)C)O)C)NC1
0 C[C@]12CCCC([C@@H]1CC[C@@]3([C@@H]2CC(=O)O3)C)(C)C
0 C[C@]12CCC(=O)C=C1CC[C@@H]3[C@@]2([C@H](C[C@]4([C@H]3C[C@@H]5[C@]4(OC(O5)(C)C)C(=O)CCl)C)O)F
0 CC(=O)OCC(=O)[C@@]12[C@@H](C[C@@H]3[C@@]1(C[C@@H]([C@]4([C@H]3CCC5=CC(=O)C=C[C@@]54C)F)O)C)OC6(O2)CCCC6
0 CC1=C(C(CCC1=O)(C)C)/C=C/C(=C/C=C/C(=C/C=C/C=C(/C=C/C=C(/C=C/C2=C(C(=O)CCC2(C)C)C)\C)\C)/C)/C
0 CC1=C(C(C[C@@H](C1)O)(C)C)/C=C/C(=C/C=C/C(=C/C=C/C=C(\C)/C=C/C=C(\C)/C=C/[C@H]2C(=C[C@@H](CC2(C)C)O)C)/C)/C

0 C[C@@H]1CC[C@@H]2[C@@]13C[C@H](C2(C)C)C(=C(C3)C(=O)C)C
0 C[C@]12CC[C@H]3[C@H]([C@@H]1CC[C@@H]2C(=O)NC(C)(C)C)CC=C4[C@@]3(CCC(=C4)C(=O)O)C
0 CC(C)C1=CC2=CC[C@@H]3[C@@]([C@H]2CC1)(CCC[C@@]3(C)C(=O)OC)C
0 C[C@H]1/C=C/C=C/2\CO[C@H]3[C@@]2([C@@H](C=C([C@H]3O)C)C(=O)O[C@H]4C[C@@H](C/C=C(/[C@H]1O[C@H]5C[C@@H]([C@H]([C@@H](O5)C)O[C@H]6C[C@@H]([C@H]([C@@H](O6)C)O)OC)OC)\C)O[C@]7(C4)C=C[C@@H]([C@H](O7)C8CCCCC8)C)O
0 C[C@]12C[C@@H]([C@]3([C@H]([C@@H]1C[C@@H]4[C@]2(OC(O4)(C)C)C(=O)COC(=O)CC(C)(C)C)CCC5=CC(=O)C=C[C@@]53C)F)O
0 C[C@@]12CC[C@@H](C1(C)C)CC2=O
0 C[C@H]1CC[C@@H]([C@H](C1)O)C(C)C
0 CC1CCC(C(C1)O)C(C)C
0 CC1(C2CCC1(C(=O)C2)C)C
0 C[C@@H]1CC[C@H]([C@@H](C1)O)C(C)C
0 CC1=CCC(CC1)C(C)(C)O
0 CC12CCCC=C1C(=O)OC2=O
0 CC1=C(C(CCC1)(C)C)/C=C/C(=C/C=C/C(=C/C(=O)OC2=C(C3=C(C(=C2C)C)O[C@](CC3)(C)CCC[C@H](C)CCC[C@H](C)CCCC(C)C)C)/C)/C
0 CC1=C(C=C2CC[C@@](OC2=C1C)(C)CCC[C@H](C)CCC[C@H](C)CCCC(C)C)O
0 CCCCCCCCCCCCCCCC(=O)O[C@H]1CC(C(=C(C1)C)/C=C/C(=C/C=C/C(=C/C=C/C=C(/C=C/C=C(/C=C/C2=C(C[C@H](CC2(C)C)OC(=O)CCCCCCCCCCCCCCC)C)\C)\C)/C)/C)(C)C
0 CC1=C(C2=C(CC[C@@](O2)(C)CCC[C@H](C)CCC[C@H](C)CCCC(C)C)C(=C1O)C)C
0 C[C@]12CCC(=O)C=C1C[C@H]([C@@H]3[C@]24[C@H](O4)C[C@]5([C@H]3CC[C@@]56CCC(=O)O6)C)C(=O)OC
0 CC1=C(C(=O)C2=CC=CC=C2C1=O)C/C=C(\C)/CC/C=C(\C)/CC/C=C(\C)/CCC=C(C)C
0 CCCCC(C)(C/C=C/[C@H]1[C@@H](CC(=O)[C@@H]1CCCCCCC(=O)OC)O)O
0 CC1=C(C(=O)C2=CC=CC=C2C1=O)C/C=C(\C)/CCC[C@H](C)CCC[C@H](C)CCCC(C)C

0 C[C@@H]1CC2(C3C(O3)(C(O2)O)C)OC4C1[C@]5(CC[C@@]67C[C@@]68CCC(C([C@@H]8CC=C7[C@@]5(C4)C)(C)C)O[C@H]9[C@@H]([C@H]([C@@H](CO9)O)O)O)C
0 CCC(=O)O[C@@H]1C[C@H]2CC[C@@]1(C2(C)C)C
0 C[C@]12C[C@@H]([C@]3([C@H]([C@@H]1C[C@@H]4[C@]2(OC(O4)(C)C)C(=O)CO)CCC5=CC(=O)C=C[C@@]53C)F)O
0 C[C@]12CCC(=O)C=C1[C@H](C[C@@H]3[C@@H]2[C@H](C[C@]4([C@H]3C[C@@H]5[C@]4(OC(O5)(C)C)C(=O)CO)C)O)F
1 C[C@]12CC[C@H]3[C@H]([C@@H]1CC[C@@H]2O)CC[C@@H]4[C@@]3(C=C[C@@H](C4)O)C
1 C[C@]12CC[C@H]3[C@H]([C@@H]1CCC2=O)CC[C@@H]4[C@@]3(C=CC(=O)C4)C
1 C[C@]12CC[C@H]3[C@H]([C@@H]1CCC2=O)CC[C@@H]4[C@@]3(C=C[C@@H](C4)O)C
1 C[C@]12CC[C@H]3[C@H]([C@@H]1CC[C@@H]2O)CC[C@@H]4[C@@]3(C=CC(=O)C4)C
1 C[C@]12CC=CCC1CC[C@@H]3[C@@H]2CC[C@]4([C@H]3CCC4)CO
1 C[C@]12CC[C@H]3[C@H]([C@@H]1CCC2=O)CC[C@@H]4[C@@]3(CC=CC4)C
1 C[C@]12CCC=CC1CC[C@@H]3[C@@H]2CC[C@]4([C@H]3CCC4)CO
1 C[C@]12CC[C@H]3[C@H]([C@@H]1CC[C@@H]2O)CCC4=C[C@H](CC[C@]34C)O
1 C[C@]12CC[C@H]3[C@H]([C@@H]1CC[C@@H]2O)CCC4=C(C(=O)CC[C@]34C)O
1 C[C@]12CC[C@H]3[C@H]([C@@H]1CCC2=O)CC=C4[C@@]3(CCC(=O)C4)C
1 C[C@]12CC[C@H]3[C@H]([C@@H]1CCC2=O)C(=O)C=C4[C@@]3(CC[C@@H](C4)O)C
1 C[C@]12CC[C@H]3[C@H]([C@@H]1CCC2=O)[C@H](C=C4[C@@]3(CC[C@@H](C4)O)C)O
1 C[C@]12CC[C@H]3[C@H]([C@@H]1CC[C@@H]2O)CCC4=C[C@H](CC[C@H]34)O
1 C[C@]12CC[C@H]3[C@H]([C@@H]1CCC2=O)CCC4=CC(=O)CC[C@H]34
1 C[C@]12CC[C@@H]3[C@H]4CC[C@H](C[C@@H]4CC[C@H]3[C@@H]1CCC2=O)O
1 C[C@]12CC[C@@H]3[C@H]4CC[C@H](C[C@H]4CC[C@H]3[C@@H]1CCC2=O)O
1 C[C@]12CCC(=O)C[C@@H]1CC[C@@H]3[C@@H]2CC[C@]4([C@H]3CC[C@@H]4O)C
1 C[C@]12CC[C@H]3[C@H]([C@@H]1CC[C@@H]2O)CC=C4[C@@]3(CC[C@@H](C4)O)C
1 C[C@]12CCC(=O)C=C1CC[C@@H]3[C@@H]2CC[C@]4([C@H]3CCC4=O)C
1 C[C@]12CC[C@H](C[C@@H]1CC[C@@H]3[C@@H]2CC[C@]4([C@H]3CCC4=O)C)O
1 C[C@@H]1CC2=CC(=O)CC[C@@]2([C@@H]3[C@@H]1[C@@H]4CC[C@]([C@]4(CC3)C)(C)O)C

1 C[C@]12CC[C@H]3[C@H]([C@@H]1CC[C@@H]2O)CCC4=CC(=O)C=C[C@]34C
1 C[C@]12CC[C@H]3[C@H]([C@@H]1CCC2=O)CCC4=CC(=O)C=C[C@]34C
1 C[C@H]1CC2=CC(=O)CC[C@@]2([C@@H]3[C@@H]1[C@@H]4CC[C@]([C@]4(CC3)C)(C)O)C
1 C[C@]12CC[C@H]3[C@H]([C@@H]1CC[C@@H]2O)CCC4=C(C(=O)CC[C@]34C)Cl
1 C[C@]12CC[C@H]3[C@H]([C@@H]1CC[C@]2(C#C)O)CCC4=CC5=C(C[C@]34C)C=NO5
1 C[C@]12CC[C@H]3[C@H]([C@@H]1CC[C@]2(C)O)CCC4=C(C(=O)C=C[C@]34C)Cl
1 C[C@]12CC[C@H]3[C@H]([C@@H]1CC[C@]2(C)O)CC[C@@H]4[C@@]3(CC=CC4)C
1 C[C@@H]1C[C@]2([C@@H](CC[C@@H]3[C@@H]2CC[C@]4([C@H]3CC[C@@H]4O)C)CC1=O)C
1 C[C@]12CC[C@@H](C[C@@H]1CC[C@@H]3[C@@H]2CC[C@]4([C@H]3CCC4=O)C)O
1 C[C@]12CCC(=O)C[C@@H]1CC[C@@H]3[C@@H]2CC[C@]4([C@H]3CC[C@H]4O)C
1 C[C@]12CC[C@H]3[C@H]([C@@H]1CC[C@H]2O)CCC4=CC(=O)CC[C@]34C
1 CC[C@@]1(CC[C@@H]2[C@@]1(CC[C@H]3[C@H]2CCC4=CCCC[C@H]34)C)O
1 C[C@]12CC[C@H](C[C@H]1CC[C@@H]3[C@@H]2CC[C@]4([C@H]3CCC4=O)C)O
1 C[C@]12CCC(=O)C=C1CC[C@@H]3[C@@]2([C@H](C[C@]4([C@H]3CC[C@]4(C)O)C)O)F
1 C[C@@]1(CC[C@@H]2[C@@]1(C[C@H]([C@H]3[C@H]2CCC4=CC(=O)C(=C[C@]34C)C=O)O)C)O
1 C[C@]12CC[C@H]3[C@H]([C@@H]1CC[C@]2(C)O)CC[C@@H]4[C@@]3(CC5=NON=C5C4)C
1 CC[C@]12C=CC3=C4CCC(=O)C=C4CC[C@H]3[C@@H]1CC[C@]2(C#C)O
1 C1C[C@@H](N(C1)C2=CC(=C(C=C2)C#N)C(F)(F)F)[C@H](C(F)(F)F)O
1 C[C@]12CCC(=O)C[C@@H]1CC[C@@H]3[C@@H]2CC[C@]4([C@H]3CC[C@]4(C)O)C
1 C[C@H]1CC(=O)C[C@H]2[C@]1([C@H]3CC[C@]4([C@H]([C@@H]3CC2)CC[C@@H]4O)C)C
1 C[C@]12CC[C@H]3[C@H]([C@@H]1CC[C@]2(C)O)CCC4=CC(=O)C=C[C@]34C
1 CC1=CC(=O)C[C@H]2[C@]1([C@H]3CC[C@]4([C@H]([C@@H]3CC2)CC[C@@H]4O)C)C
1 C[C@]12CC[C@@H](CC1=CC[C@@H]3[C@@H]2CC[C@]4([C@H]3CC[C@]4(C)O)C)O
1 C[C@@H]1C[C@]2([C@@H](CC[C@@H]3[C@@H]2CC[C@]4([C@H]3CC[C@]4(C)O)C)CC1=O)C
1 C[C@]12CC[C@H]3[C@H]([C@@H]1CC[C@]2(C)O)CC[C@@H]4[C@@]3(C=CC(=O)C4)C
1 C[C@]12CCC(=O)C(=C1CC[C@@H]3[C@@H]2CC[C@]4([C@H]3CC[C@]4(C)O)C)Cl

1	C[C@]12CCC3=C4CCC(=O)C=C4CC[C@H]3[C@@H]1CC[C@]2(C)O
1	C[C@]12CC[C@H]3[C@H]([C@@H]1CC[C@]2(C)O)CCC4=CC(=O)CC[C@H]34
1	C[C@]12CCC(=O)C=C1CC[C@@H]3[C@@H]2CC[C@]4([C@H]3CC[C@]4(C)O)C
1	C[C@@]1(CC[C@@H]2[C@@]1(C=CC3=C4CCC(=O)C=C4CC[C@@H]23)C)O
1	C[C@@H]1CC2=CC(=O)CC[C@@H]2[C@@H]3[C@@H]1[C@@H]4CC[C@]([C@]4(CC3)C)(C)O
1	C[C@]12CC[C@H]3[C@H]([C@@H]1CC[C@@H]2O)CCC4=CC(=O)CC[C@H]34
1	CC[C@]12CC[C@H]3[C@H]([C@@H]1CC[C@]2(CC)O)CCC4=CC(=O)CC[C@H]34
1	C[C@]12CC[C@H]3[C@H]([C@@H]1CC[C@@H]2O)CCC4=C(C(=O)CC[C@H]34)Cl
1	CC[C@@]1(CC[C@@H]2[C@@]1(CC[C@H]3[C@H]2CCC4=CC(=O)CC[C@H]34)C)O
1	C[C@]12CC[C@H]3[C@H]([C@@H]1CC[C@@H]2O)CCC4=C(C(=O)CC[C@H]34)O
1	C[C@]12CC[C@H]3[C@H]([C@@H]1CC[C@]2(C)O)CC[C@@H]4[C@@]3(COC(=O)C4)C
1	C[C@]12CCC(=O)C(=C1CC[C@@H]3[C@@H]2CC[C@]4([C@H]3CC[C@]4(C)O)C)O
1	C[C@]12CC[C@H]3[C@H]([C@@H]1CC[C@]2(C)O)CC[C@@H]4[C@@]3(C/C(=C/O)/C(=O)C4)C
1	C[C@]12CC[C@H]3[C@H]([C@@H]1CCC2=O)CC=C4[C@@]3(CC[C@@H](C4)O)C
1	C[C@]12CC[C@H]3[C@H]([C@@H]1CC[C@@H]2OC4CCCCO4)CC[C@@H]5[C@@]3(CC6=C(C5)NN=C6)C
1	C[C@]12CC[C@H]3[C@H]([C@@H]1CC[C@@H]2OC4=CCCC4)CCC5=CC(=O)C=C[C@]35C
1	CC1=C(C=CC(=C1Cl)C#N)N[C@@H](C2=NN=C(O2)C3=CC=C(C=C3)C#N)[C@H](C)O
1	C[C@]12CC[C@H]3[C@H]([C@@H]1CC[C@]2(C)O)CC[C@@H]4[C@@]3(CC5=C(C4)NN=C5)C
1	CC1=C[C@]2([C@@H](CC[C@@H]3[C@@H]2CC[C@]4([C@H]3CC[C@@H]4O)C)CC1=O)C
1	C[C@]12CC[C@H]3[C@H]([C@@H]1CC[C@@H]2O)CCC4=CC(=O)CC[C@]34C
1	CC[C@@]1(CC[C@@H]2[C@@]1(C=CC3=C4CCC(=O)C=C4CC[C@@H]23)CC)O
1	C[C@@H]1CC2=C(CCC(=O)C2)[C@@H]3[C@@H]1[C@@H]4CC[C@]([C@]4(CC3)C)(C#C)O
1	C[C@]12C=CC3=C4CCC(=O)C=C4CC[C@H]3[C@@H]1CC[C@@H]2O
1	C[C@H]1CCC[C@@H](CCCCCC2=C(C(=CC(=C2)O)O)C(=O)O1)O
1	CC(C)N[C@@H]1CCN2C3=C([C@H]1O)C=CC=C3NC2=O

Note: 1, positive chemical; 0, negative chemical

Table S2. Number of chemicals in the training and test

	Positive	Negative	Total
Training set	46	83	129
Test set	26	30	56
Total	72	113	185

Table S3. The optimal hyperparameters of three algorithms in six models.

Molecular descriptors	Algorithms	Hyperparameters	Molecular descriptors	Algorithms	Hyperparameters
	RF	max_depth=30, n_estimators=101		RF	max_depth=30, n_estimators=101
RDKit	SVM	gamma=0.001, C=2.0, cache_size=5000	Pychem	SVM	gamma=0.001, C=2.0, cache_size=5000
	ANN	alpha=0.1, hidden_layer_sizes=12		ANN	alpha=0.1, hidden_layer_sizes=12

Note: SVM, support vector machine; RF, random forests; ANN, artificial neural networks.

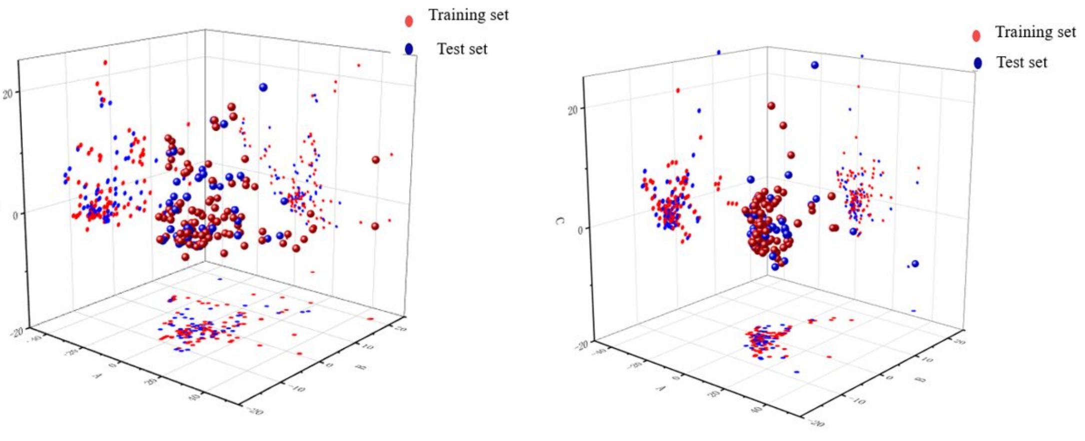

Figure S2. Chemicals spatial distribution.

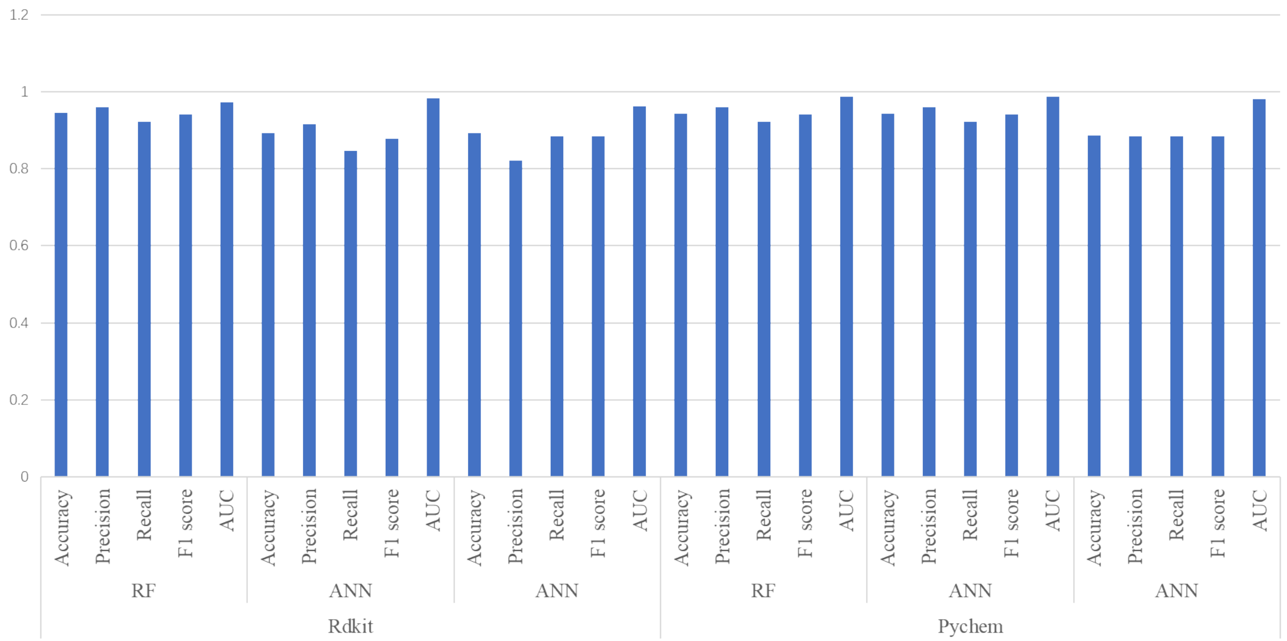
1.2
1
0.8
0.6
0.4
0.2
0
Accuracy
Precision
Recall
F1 score
AUC
RF
ANN
ANN
Rdkit
Pychem

Figure S3. Performance of validation for the models. RF, Random Forests; ANN, Artificial Neural Networks; SVM, Support Vector Machine; AUC, Area Under the Receiver Operating Characteristic Curve.

Table S4. The prediction results of active components of herbal medicines driven by machine learning models

			RDkit			Pychem		
	Molecule Name	CID	RF	ANN	SVM	RF	ANN	SVM
Radix Astragali	vanillic acid	8468	0	1	0	0	0	0
Radix Astragali	Heriguard	1794427	0	1	0	0	1	0
Radix Astragali	Jaranol	5318869	1	1	0	0	1	1
Radix Astragali	Rhamnocitrin	5320946	1	1	0	0	1	1
Radix Astragali	isorhamnetin	5281654	1	1	0	0	1	1
Radix Astragali	3,9-di-O-methylnissolin	15689655	1	0	0	0	0	0
Radix Astragali	7-O-methylisomucronulatol	15689652	1	0	0	0	0	0
Radix Astragali	Arabinose,d	66308	0	1	1	0	0	0
Radix Astragali	D-Galacturonic acid, homopolymer	84740	0	1	0	0	0	0
Radix Astragali	DL-Glucuronic acid	65041	0	1	0	0	0	0
Radix Astragali	Fucopyranose, L-	3034656	0	1	1	0	0	0
Radix Astragali	daidzein	5281708	0	1	1	0	1	1
Radix Astragali	formononetin	5280378	0	1	0	0	1	1
Radix Astragali	Soyasaponin I	112097	0	1	1	0	0	0
Radix Astragali	Flavaxin	493570	0	1	0	0	1	0
Radix Astragali	Mucronulatol	442811	1	1	0	0	0	0
Radix Astragali	Caffeate	4484949	1	1	0	0	0	0
Radix Astragali	Calycosin	5280448	1	1	0	0	1	1
Radix Astragali	3'-Hydroxy-4'-methoxyisoflavone-7-O-beta-D-glucoside	5318267	0	1	0	0	1	0
Radix Astragali	XLS	644160	0	1	1	0	0	0
Radix Astragali	nicotinic acid	938	0	1	0	0	0	0
Radix Astragali	kaempferol	5280863	1	1	0	0	1	1
Radix Astragali	RAM	439710	0	1	1	0	0	0
Radix Astragali	coumarin	323	0	1	0	0	0	0
Radix Astragali	FA	135398658	0	1	0	0	0	0
Radix Astragali	Hirsutrin	5280804	0	1	0	0	1	0
Radix Astragali	(3R)-3-(2-hydroxy-3,4-	602152	1	1	0	0	0	0

	dimethoxyphenyl)chroman-7-ol							
Radix Astragali	LUPENONE	92158	0	0	1	0	0	0
Radix Astragali	1,7-Dihydroxy-3,9-dimethoxy pterocarpene	5316760	1	1	0	0	1	0
Radix Astragali	quercetin	5280343	1	1	0	0	1	1
Panax Guinquefolius	heptadeca-1,8-dien-4,6-diyn-3,10-diol	6438621	0	1	0	0	0	0
Panax Guinquefolius	Chrysosplenetin	5281608	0	1	0	0	0	0
Panax Guinquefolius	(3S,8E,10S)-heptadeca-1,8-dien-4,6-diyne-3,10-diol	5318010	0	1	0	0	0	0
Panax Guinquefolius	Lindenene	72723223	0	0	1	0	0	0
Panax Guinquefolius	longifolene	1796220	0	0	1	0	0	0
Panax Guinquefolius	panaxytriol	93484	0	1	0	0	0	0
Panax Guinquefolius	Phlegmariuine-N	195484	0	0	0	0	1	0
Panax Guinquefolius	(1R,3R,5R)-6,6-dimethyl-2-methylene-3-norpinanol	102667	0	0	1	0	0	0
Panax Guinquefolius	vitamin b6	1054	0	1	0	0	1	0
Panax Guinquefolius	Sorbin	6904	0	1	0	0	0	0
Panax Guinquefolius	(8S,9S,10R,13R,14S,17R)-17-[(1R,4R)-4-ethyl-1,5-dimethylhexyl]-10,13-dimethyl-1,2,8,9,11,12,14,15,16,17-decahydrocyclopenta[a]phenanthren-7-one	12444466	0	0	1	0	0	0

Panax Guinquefolius	vitamin a	445354	0	0	1	0	0	0
Panax Guinquefolius	α-cedrene	6431015	0	0	1	0	0	0
Panax Guinquefolius	2-(4-cyclohexylphenoxy)ethanol	160168	0	0	1	0	0	0
Panax Guinquefolius	20-(R)-Ginsenoside-Rg2_qt	157711	0	0	0	0	0	0
Panax Guinquefolius	3-Methoxyherbacetin	5319442	1	1	0	1	1	1
Panax Guinquefolius	Isocaryophyllene	5281522	0	0	1	0	0	0
Panax Guinquefolius	(Z)-caryophyllene	6429301	0	0	1	0	0	0
Panax Guinquefolius	panaxynol	5281149	0	1	0	0	0	0
Panax Guinquefolius	Pulegone	442495	0	0	1	0	0	0
Panax Guinquefolius	capsaicin	1548943	0	1	0	0	0	0
Panax Guinquefolius	beta-caryophyllene	5281515	0	0	1	0	0	0
Panax Guinquefolius	Encecalin	114703	0	0	1	0	1	1
Panax Guinquefolius	16-Oxoseratenediol	5320337	0	0	1	0	0	0
Panax Guinquefolius	Methose	5984	0	1	0	0	0	0
Panax Guinquefolius	panaxydol	126312	0	1	0	0	0	0
Panax Guinquefolius	Hepanal	15560276	0	0	1	0	0	0
Codonopsis pilosula	poriferasta-7,22E-dien-3beta-ol	5283663	0	0	1	0	0	0
Codonopsis pilosula	2-methoxyfuranodiene	6325622	0	0	0	0	1	1
Codonopsis pilosula	(-)-alpha-Pinene	440968	0	0	1	0	0	0

Codonopsis pilosula	(+/-)-Isoborneol	6321405	0	0	1	0	0	0
Codonopsis pilosula	Perlolyrine	160179	0	1	0	0	1	0
Codonopsis pilosula	delta-Guaiene	94275	0	0	1	0	0	0
Codonopsis pilosula	capsaicin	1548943	0	1	0	0	0	0
Codonopsis pilosula	Syringaldehyde	8655	0	1	0	0	0	0
Codonopsis pilosula	Stigmasterol	5280794	0	0	1	0	0	0
Codonopsis pilosula	Syrigin	5316860	0	1	0	0	0	0
Codonopsis pilosula	D-Friedoolean-14-en-3-one	92785	0	0	1	0	0	0
Codonopsis pilosula	Shekanin	5281810	0	1	0	0	1	0
Codonopsis pilosula	tectorigenin	5281811	1	1	0	0	1	1
Codonopsis pilosula	7-Methoxy-2-methyl isoflavone	354368	0	0	0	0	1	0
Codonopsis pilosula	nicotinic acid	938	0	1	0	0	0	0
Codonopsis pilosula	Spinasterol	5281331	0	0	1	0	0	0
Codonopsis pilosula	atractylenolideII	14448070	0	0	1	0	0	0
Codonopsis pilosula	atractylenolide iii	155948	0	0	1	0	1	0
Codonopsis pilosula	Encecalin	114703	0	0	1	0	1	1
Codonopsis pilosula	Frutinone A	441965	0	1	0	0	1	0
Codonopsis pilosula	FOA	6919	0	1	0	0	0	0
Codonopsis pilosula	luteolin	5280445	1	1	1	1	1	1
Codonopsis pilosula	Norharman	64961	0	1	0	0	1	0
Codonopsis pilosula	3-beta-Hydroxymethyllenetanshiquino	134165	1	1	1	0	0	0

	ne							
Codonopsis pilosula	HMF	237332	0	1	0	0	0	0
Codonopsis pilosula	apigenin	5280443	0	1	0	0	1	1
Codonopsis pilosula	EA-fructofuranoside	133590	0	1	0	0	0	0
Codonopsis pilosula	Galuteolin	5280637	0	1	0	0	1	0
Codonopsis pilosula	o-(o-Methoxyphenoxy)phenol	572803	0	1	0	0	0	0
Codonopsis pilosula	3-METHYLCARBAZOLE	20746	0	1	0	0	1	0
Codonopsis pilosula	L-Sulforaphane	9577379	0	1	0	0	0	0
Codonopsis pilosula	4-Phenylbicyclo[2,2,2]octan-1-ol	327096	0	1	1	0	1	0
Codonopsis pilosula	Codonopsine	442631	1	1	0	0	0	0
Codonopsis pilosula	Coelogin	442697	1	1	0	0	0	0
Codonopsis pilosula	Ethyl-p-digallate	5317260	0	1	0	0	1	0
Codonopsis pilosula	fritillaziebinol	5317397	1	0	0	1	1	0
Codonopsis pilosula	glycitein	5317750	1	1	0	0	1	1
Codonopsis pilosula	BHG	181215	0	1	1	0	0	0
Codonopsis pilosula	(8S,9S,10R,13R,14S,17R)-17-[(E,2R,5S)-5-ethyl-6-methylhept-3-en-2-yl]-10,13-dimethyl-1,2,4,7,8,9,11,12,14,15,16,17-dodecahydrocyclopenta[a]phen	14807783	0	0	1	0	0	0

	anthren-3-one							
Codonopsis pilosula	alpha-Stigmasta-7,22-dien-3-one	91692437	0	0	1	0	0	0
Codonopsis pilosula	Codopiloic acid	178371	0	1	0	0	0	0
Codonopsis pilosula	ethyl-β-D-fructofuranoside	11769694	0	1	0	0	0	0
Codonopsis pilosula	luteolin-7-o-glucoside	5280637	0	1	0	0	1	0
Codonopsis pilosula	()-beta-Pinene	10290825	0	0	1	0	0	0
Angelica sinensis	(-)-alpha-Pinene	440968	0	0	1	0	0	0
Angelica sinensis	Verbenone	29205	1	1	0	0	0	0
Angelica sinensis	(+)-Ledol	11074994	0	1	1	0	0	0
Angelica sinensis	beta-Chamigrene	442353	0	0	1	0	0	0
Angelica sinensis	adenine	190	0	1	0	0	1	0
Angelica sinensis	cis-Thujopsene	442402	0	0	1	0	0	0
Angelica sinensis	3-Butylidene-7-hydroxyphthalide	5281559	0	1	0	0	0	1
Angelica sinensis	senkyunolide-C	642374	0	1	0	0	0	0
Angelica sinensis	senkyunolide-D	11264524	0	1	0	0	0	0
Angelica sinensis	senkyunolide-E	11830530	0	1	0	0	0	0
Angelica sinensis	cis-ligustilide	5319022	0	1	0	0	0	0
Angelica sinensis	Guasol	460	0	1	0	0	0	0
Angelica sinensis	beta-Selinene	28237	0	0	1	0	0	0
Angelica sinensis	CADINENE	3032853	0	0	1	0	0	0
Angelica sinensis	Acoradiene	90351	0	0	1	0	0	0

Angelica sinensis	D-Galacturonic acid, homopolymer	84740	0	1	0	0	0	0
Angelica sinensis	Scopoletol	5280460	0	1	0	0	1	0
Angelica sinensis	nicotinic acid	938	0	1	0	0	0	0
Angelica sinensis	Maruzen M	32758	0	1	0	0	1	0
Angelica sinensis	Eucarvone	136330	0	1	1	0	0	0
Angelica sinensis	(1S,4aR,8aR)-1-isopropyl-7-methyl-4-methylene-2,3,4a,5,6,8a-hexahydro-1H-naphthalene	6432404	0	0	1	0	0	0
Angelica sinensis	Stigmasterol	5280794	0	0	1	0	0	0
Angelica sinensis	vanillin	1183	0	1	0	0	0	0
Angelica sinensis	bicycloelemene	56842786	0	0	1	0	0	0
Angelica sinensis	WLN: VH6	6990	0	1	0	0	0	0
Angelica sinensis	phosphatdic acid	25163993	0	1	0	0	0	0
Angelica sinensis	phosphatidylinositol	99852308	0	1	0	0	0	0
Angelica sinensis	ESEN	6811	0	1	0	0	0	0
Angelica sinensis	sedanolide	5018391	0	1	0	0	0	0
Angelica sinensis	senkyunolide	3085257	0	1	0	0	0	0
Angelica sinensis	α-acoradiene	6428281	0	0	1	0	0	0
Angelica sinensis	InChI=1/C15H24/c1-10-7-8-15-9-12(10)14(3,4)13(15)6-5-11(15)2/h7,11-	6431015	0	0	1	0	0	0

	13H,5-6,8-9H2,1-4H							
Angelica sinensis	α-copaene	50919054	0	1	1	0	0	0
Angelica sinensis	(1R,4R,5S)-4-isopropenyl-1,8-dimethylspiro[4.5]dec-8-ene	13743810	0	0	1	0	0	0
Angelica sinensis	1,1,5-trimethyl-2-formylcyclohexa-2,5-diene-4-one	129716150	0	1	1	0	0	0
Angelica sinensis	5-Indolol	16054	0	1	0	0	0	0
Angelica sinensis	aromadendrene	10899740	0	0	1	0	0	0
Angelica sinensis	(3E)-3-butylidene-7-hydroxy-2-benzofuran-1-one	5852943	0	1	0	0	0	1
Angelica sinensis	Coniferyl ferulate	6441913	1	0	0	0	0	0
Angelica sinensis	bergamotene	521569	0	0	1	0	0	0
Fructus Lycii	copaene	19725	0	1	1	0	0	0
Fructus Lycii	citric acid	311	0	0	0	0	1	0
Fructus Lycii	vitamin c	54670067	0	1	0	0	0	0
Fructus Lycii	Scopolin	439514	0	1	0	0	1	0
Fructus Lycii	TAU	1123	0	1	0	0	1	0
Fructus Lycii	beta-Ionone	638014	0	0	1	0	0	0
Fructus Lycii	Cumalic acid	68141	0	1	0	0	1	0
Fructus Lycii	alpha cadinene	12306048	0	0	1	0	0	0
Fructus Lycii	Stigmasterol	5280794	0	0	1	0	0	0
Fructus Lycii	PEY	995	0	0	0	0	1	0
Fructus Lycii	Scopoletol	5280460	0	1	0	0	1	0
Fructus Lycii	nicotinic acid	938	0	1	0	0	0	0
Fructus Lycii	Physcion	10639	0	1	0	0	1	1
Fructus Lycii	campesterol	173183	0	0	1	0	0	0
Fructus Lycii	Ostreasterol	92113	0	0	1	0	0	0
Fructus Lycii	delta-amorphene	441005	0	0	1	0	0	0

Fructus Lycii	Safranal	61041	0	0	1	0	0	0
Fructus Lycii	24-methylidenelophenol	5283640	0	0	1	0	0	0
Fructus Lycii	19435-97-3	3084311	0	1	1	0	0	1
Fructus Lycii	glycitein	5317750	1	1	0	0	1	1
Fructus Lycii	paeonol	11092	0	1	0	0	0	0
Fructus Lycii	Cedrol	65575	0	1	1	0	0	0
Fructus Lycii	CLR	5997	0	0	1	0	0	0
Fructus Lycii	WLN: E6E	12368	0	1	0	0	0	0
Fructus Lycii	Stearyl iodide	12402	0	1	0	0	0	0
Fructus Lycii	24-ethylcholesta-5,22-dienol	69473919	0	0	1	0	0	0
Fructus Lycii	Fucosterol	5281326	0	0	1	0	0	0
Fructus Lycii	2-o-(beta-d-glucopyranosyl)-ascorbic acid	54706833	0	1	0	0	1	0
Fructus Lycii	31-norlanosterol	102179328	0	0	1	0	0	0
Fructus Lycii	1-(2-hydrazino-4-methyl-5-pyrimidinyl)ethanone	341594	0	1	0	0	1	0
Fructus Lycii	Lophenol	160482	0	0	1	0	0	0
Fructus Lycii	4alpha,24-dimethylcholesta-7,24-dienol	129726843	0	0	1	0	0	0
Fructus Lycii	Maaliol	10944069	0	1	1	0	0	0
Fructus Lycii	beta-Cholestanol	6665	0	0	1	0	0	0
Fructus Lycii	lathosterol	65728	0	0	1	0	0	0
Fructus Lycii	octahydro-4,4,8,8-tetramethyl-4a,7-methano-4aH-naphth[1,8a-b]oxirene	107035	0	1	0	0	1	0
Fructus Lycii	Solavetivone	442399	0	1	1	0	0	0
Fructus Lycii	quercetin	5280343	1	1	0	0	1	1
Ganoderma Lucidum	1,8-Cineole	2758	0	0	1	0	0	0
Ganoderma	campesta-	5283669	0	0	1	0	0	0

Lucidum	7,22E-dien-3beta-ol							
Ganoderma Lucidum	5alpha-Lanosta-7,9(11),24-triene-15alpha,26-dihydroxy-3-one	21635716	0	0	1	0	0	0
Ganoderma Lucidum	alpha-D-Arabinofuranosyladenine	96368	0	1	0	0	1	0
Ganoderma Lucidum	alpha-Terpineol	17100	0	0	1	0	0	0
Ganoderma Lucidum	bergamotene (Z,.alpha.,cis)	6429302	0	0	1	0	0	0
Ganoderma Lucidum	beta-Bazzanene	12444487	0	0	1	0	0	0
Ganoderma Lucidum	beta-Irone	5375215	0	0	1	0	0	0
Ganoderma Lucidum	Bisabolol oxide B	117301	0	1	1	0	0	1
Ganoderma Lucidum	Daucene	177773	0	0	1	0	0	0
Ganoderma Lucidum	Ergosta-7,22-dien-3-one	99511	0	0	1	0	0	0
Ganoderma Lucidum	Peroxyergosterol	5351516	0	0	1	0	1	0
Ganoderma Lucidum	(E,6R)-2-methyl-6-[(5R,10S,13R,14R,17R)-4,4,10,13,14-pentamethyl-3-oxo-1,2,5,6,12,15,16,17-octahydrocyclopenta[a]phenanthren-17-yl]hept-2-enal	13934282	0	0	1	0	0	0
Ganoderma Lucidum	ganoderal B	14015440	0	0	1	0	0	0
Ganoderma Lucidum	Ganoderic aldehyde A	14484704	0	0	1	0	0	0

Ganoderma Lucidum	Ganoderiol F	471008	0	0	1	0	0	0
Ganoderma Lucidum	ganodermadiol	13934285	0	0	1	0	0	0
Ganoderma Lucidum	ganodermenonol	6439006	0	0	1	0	0	0
Ganoderma Lucidum	(5R,10S,13R,14R,17R)-17-[(E,2R)-7-hydroxy-6-methylhept-5-en-2-yl]-4,4,10,13,14-pentamethyl-1,2,5,6,12,15,16,17-octahydrocyclopenta[a]phenanthren-3-one	13934284	0	0	1	0	0	0
Ganoderma Lucidum	GCS	441477	0	1	0	0	0	0
Ganoderma Lucidum	Isobazzanene	14830703	0	0	1	0	0	0
Ganoderma Lucidum	Lucialdehyde A	11048424	0	0	1	0	0	0
Ganoderma Lucidum	Lucialdehyde C	10366713	0	0	1	0	0	0
Ganoderma Lucidum	lucidone A	71453988	0	0	1	0	1	1
Ganoderma Lucidum	lucidone B	14109411	0	0	0	0	1	0
Ganoderma Lucidum	lucidone C	14109415	0	0	1	0	1	1
Ganoderma Lucidum	dl-Thujone	261491	0	1	1	0	0	0
Ganoderma Lucidum	(-)-alpha-Pinene	440968	0	0	1	0	0	0
Ganoderma Lucidum	copaene	12303902	0	1	1	0	0	0
Ganoderma Lucidum	(s)-carvone	16724	0	0	1	0	0	0
Ganoderma Lucidum	l-carvone	439570	0	0	1	0	0	0
Ganoderma	Cerevisterol	10181133	0	0	1	0	0	0

Lucidum								
Ganoderma Lucidum	ergosterol	444679	0	0	1	0	0	0
Ganoderma Lucidum	MTL	6251	0	1	0	0	0	0
Ganoderma Lucidum	delta-amorphene	10223	0	0	1	0	0	0
Ganoderma Lucidum	Cedrol	65575	0	1	1	0	0	0
Ganoderma Lucidum	(-)-Drimenol	3080551	0	0	1	0	0	0
Ganoderma Lucidum	(R)-p-Menth-1-en-4-ol	5325830	0	0	1	0	0	0
Rhizoma Polygonat	isoliquiritigenin	638278	0	1	0	0	1	1
Rhizoma Polygonat	DFV	114829	0	1	0	0	1	1
Rhizoma Polygonat	salicylic acid	338	0	1	0	0	0	0
Rhizoma Polygonat	baicalein	5281605	1	1	0	0	1	1
Rhizoma Polygonat	3'-Methoxydaidzein	5319422	1	1	0	0	1	1
Rhizoma Polygonat	succinic acid	21952380	0	1	0	0	0	0
Rhizoma Polygonat	(Z)-1-(2,4-dihydroxyphenyl)-3-(4-hydroxyphenyl)prop-2-en-1-one	6603886	0	1	0	0	1	1
Rhizoma Polygonat	glucuronic acid	441478	0	1	0	0	0	0
Rhizoma Polygonat	(2R)-7-hydroxy-2-(4-hydroxyphenyl)chroman-4-one	928837	0	1	0	0	1	1
Rhizoma Polygonat	GUP	439680	0	1	1	0	0	0
Rhizoma Polygonat	BGC	64689	0	1	1	0	0	0
Rhizoma Polygonat	4',5-Dihydroxyflavo	165521	0	1	0	0	1	1

	ne							
Rhizoma Polygonat	HMF	237332	0	1	0	0	0	0
Rhizoma Polygonat	apigenin	5280443	0	1	0	0	1	1
Rhizoma Polygonat	4-methylolfurfural	5318289	0	1	0	0	0	0
Rhizoma Polygonat	Oroxin A	5320313	0	1	0	0	1	0
Rhizoma Polygonat	2-Acridinecarboxylic acid	3041556	0	0	0	0	1	0
Rhizoma Polygonat	n-butyl-β-D-fructopyranoside	13059907	0	1	0	0	1	0
Rhizoma Polygonat	2-Propen-1-one, 1-(2,4-dihydroxyphenyl)-3-(4-(beta-D-glucopyranosyloxy)phenyl)-, (2E)-	5318591	0	1	0	0	0	0
Poria Cocos	Cerevisterol	10181133	0	0	1	0	0	0
Poria Cocos	Ergosterol peroxide	5351516	0	0	1	0	1	0
Poria Cocos	L-uridine	466466	0	1	1	0	1	0
Poria Cocos	ergosterol	444679	0	0	1	0	0	0
Poria Cocos	Ethyl glucoside	11127487	0	1	0	0	1	0
Pueraria	formononetin	5280378	0	1	0	0	1	1
Pueraria	daidzein	5281708	0	1	1	0	1	1
Pueraria	LUPENONE	92158	0	0	1	0	0	0
Pueraria	genistein	5280961	1	1	0	0	1	1
Pueraria	scoparone	8417	0	1	0	0	1	0
Pueraria	(R)-Allantoin	439713	0	1	0	0	1	0
Pueraria	3'-Methoxydaidzein	5319422	1	1	0	0	1	1
Pueraria	puerarin	5281807	0	1	0	0	1	0
Pueraria	7,8,4'-Trihydroxyisoflavone	5466139	0	1	1	0	1	1
Pueraria	daidzin	107971	0	1	0	0	1	0

Jujube	stepharine	98455	1	1	1	0	0	0
Jujube	Spiradine A	441756	1	0	0	0	1	0
Jujube	(4S)-4-hydroxy-4-[(E,3S)-3-hydroxybut-1-enyl]-3,5,5-trimethylcyclohex-2-en-1-one	10537120	0	1	1	0	1	0
Jujube	CMP	6131	1	1	0	0	1	0
Jujube	Daechualkaloid A	13875298	0	1	1	0	1	0
Jujube	24-Dehydrocholesterol	439577	0	0	1	0	0	0
Jujube	swertisin	124034	0	1	0	0	1	0
Jujube	1-(4-Coumaroyl)alpha-rhamnopyranose	6438942	0	1	0	0	1	0
Jujube	Asimilobine	160875	0	1	1	0	0	0
Jujube	coumestrol	5281707	0	1	0	1	1	1
Jujube	MLT	4004	0	1	0	0	0	0
Jujube	IES	802	0	1	0	0	1	0
Jujube	Sinapyl alcohol	5280507	0	1	0	0	0	0
Jujube	Vomifoliol	5280462	0	1	1	0	1	0
Jujube	Stigmasterol	5280794	0	0	1	0	0	0
Jujube	nicotinic acid	938	0	1	0	0	0	0
Jujube	(+)-catechin	9064	1	1	1	0	1	1
Jujube	Lysicamine	122691	1	0	0	0	1	0
Jujube	Methose	5984	0	1	0	0	0	0
Jujube	Vulgarin	94253	0	1	1	0	1	1
Jujube	3-[[(2S)-2,4-dihydroxy-3,3-dimethylbutanoyl]amino]propanoic acid	988	0	1	0	0	1	0
Jujube	Thiamine	1130	0	1	0	0	1	0
Jujube	Prolinum	145742	0	0	0	0	0	0
Jujube	Stepholidine	6917970	1	0	0	0	0	0
Jujube	sucrose	5988	0	1	0	0	1	0
Jujube	Moupinamide	5280537	1	1	0	0	0	0
Jujube	catechol	289	0	1	0	0	0	0

Jujube	(-)-catechin	73160	1	1	1	0	1	1
Jujube	quercetin	5280343	1	1	0	0	1	1
Jujube	GLO	107526	0	1	1	0	0	0
Liquorice	protocatechuic acid	72	0	1	0	0	0	0
Liquorice	(L)-alpha-Terpineol	443162	0	0	1	0	0	0
Liquorice	Arachic acid	10467	0	0	0	0	0	0
Liquorice	Inermine	91510	0	1	0	0	1	0
Liquorice	Vicenin-2	442664	0	1	0	0	0	0
Liquorice	Morusin	5281671	0	1	0	0	1	0
Liquorice	ICO	69867	0	1	0	0	1	0
Liquorice	isoliquiritigenin	638278	0	1	0	0	1	1
Liquorice	DFV	114829	0	1	0	0	1	1
Liquorice	Izoforon	6544	0	0	1	0	0	0
Liquorice	Glycyrol	5320083	0	1	0	0	1	0
Liquorice	Jaranol	5318869	1	1	0	0	1	1
Liquorice	Medicarpin	336327	0	0	0	0	1	1
Liquorice	oleanolic acid	10494	0	0	0	0	0	0
Liquorice	EB	7500	0	0	0	0	0	0
Liquorice	nicotiflorin	5318767	0	0	0	0	0	0
Liquorice	Pinocembrin	68071	0	1	1	0	1	1
Liquorice	butylated hydroxytoluene	31404	0	0	0	0	0	0
Liquorice	BuOH	263	0	0	0	0	0	0
Liquorice	Neouralenol	5320118	0	1	0	0	1	1
Liquorice	isorhamnetin	5281654	1	1	0	0	1	1
Liquorice	sitosterol	222284	0	0	0	0	0	0
Liquorice	Lupiwighteone	5317480	1	1	0	0	1	1
Liquorice	7,4'-Dihydroxyflavone	5282073	0	1	1	0	1	1
Liquorice	Narcissoside	5481663	0	0	0	0	0	0
Liquorice	formononetin	5280378	0	1	0	0	1	1
Liquorice	2-Caren-10-al	556516	0	1	1	0	0	0
Liquorice	Scopoletol	5280460	0	1	0	0	1	0
Liquorice	rutin	5280805	0	0	0	0	0	0
Liquorice	Calycosin	5280448	1	1	0	0	1	1
Liquorice	kaempferol	5280863	1	1	0	0	1	1
Liquorice	naringenin	932	1	1	1	1	1	1
Liquorice	Hirsutrin	5280804	0	1	0	0	1	0
Liquorice	8-Prenylwighteone	480783	0	0	0	0	1	0

Liquorice	Methylheptane	11594	0	0	0	0	0	0
Liquorice	Castanin	5281704	1	1	0	0	1	0
Liquorice	glucuronic acid	94715	0	1	0	0	0	0
Liquorice	glyasperin B	480784	0	0	0	0	0	1
Liquorice	glyasperin F	392442	0	1	0	0	1	1
Liquorice	Isotrifoliol	5318679	1	1	0	0	1	0
Liquorice	(E)-1-(2,4-dihydroxyphenyl)-3-(2,2-dimethylchromen-6-yl)prop-2-en-1-one	10881804	0	0	1	0	1	1
Liquorice	(E)-1-(2,4-dihydroxyphenyl)-3-[4-hydroxy-3-(3-methylbut-2-enyl)phenyl]prop-2-en-1-one	11099375	0	1	0	0	0	1
Liquorice	Semilicoisoflavone B	5481948	0	1	1	0	1	1
Liquorice	Glepidotin A	5281619	1	1	0	0	1	1
Liquorice	Glepidotin B	442411	1	0	0	0	1	1
Liquorice	Phaseolinisoflavan	4484952	0	0	1	0	0	1
Liquorice	3-(2-hydroxy-4-methoxyphenyl)-2H-chromen-7-ol	10378419	0	1	0	0	1	1
Liquorice	Glypallichalcone	5317768	1	1	0	0	0	0
Liquorice	echinatin	6442675	0	1	0	0	1	1
Liquorice	Liconeolignan	133867	0	1	0	0	0	0
Liquorice	Licochalcone B	5318999	1	1	0	0	1	1
Liquorice	Licoflavonol	5481964	0	1	0	0	1	1
Liquorice	Yinyanghuo D	5315396	1	1	0	0	1	1
Liquorice	Licoricone	5319013	0	1	0	0	0	0
Liquorice	Gancaonin A	5317478	0	1	0	0	1	0
Liquorice	Gancaonin B	5317479	0	1	0	0	1	0
Liquorice	Gancaonin C	6450959	0	1	0	0	1	1
Liquorice	2,3-dimethylhexane	11447	0	0	0	0	0	0
Liquorice	Prunetin	5281804	1	1	0	0	1	1
Liquorice	Gancaonin D	5317481	0	1	0	0	1	0

Liquorice	Gancaonin P	5481966	0	1	0	0	1	1
Liquorice	Gancaonin Q	480802	0	0	0	0	1	0
Liquorice	Gancaonin V	480817	0	1	1	0	0	1
Liquorice	Glycycoumarin	5317756	0	1	0	0	1	0
Liquorice	Glycyrin	480787	0	1	0	0	0	0
Liquorice	5,6,7,8-Tetrahydro-2,4-dimethylquinoline	5321849	0	0	0	0	1	0
Liquorice	(E)-1-[2,4-dihydroxy-3-(3-methylbut-2-enyl)phenyl]-3-(2,4-dihydroxyphenyl)prop-2-en-1-one	9862769	0	1	0	0	1	1
Liquorice	Licocoumarone	503731	0	1	0	0	1	0
Liquorice	Licoisoflavone	5281789	0	1	0	0	1	1
Liquorice	Licoisoflavone B	5481234	0	1	0	0	1	1
Liquorice	licoisoflavanone	392443	0	0	0	0	1	1
Liquorice	shinpterocarpin	10336244	0	0	1	0	1	1
Liquorice	glycyrrhetol	12310283	0	0	1	0	0	0
Liquorice	licopyranocoumarin	122851	0	1	0	0	1	0
Liquorice	Glyzaglabrin	5317777	1	1	0	0	1	1
Liquorice	Glabridin	124052	0	0	1	0	0	1
Liquorice	Glabranin	124049	1	0	0	0	1	1
Liquorice	Glabrene	480774	0	0	0	0	1	1
Liquorice	Glabrone	5317652	1	1	1	0	1	1
Liquorice	Eurycarpin A	5317300	1	1	1	0	1	1
Liquorice	vitexin	5280441	0	1	0	0	1	0
Liquorice	violanthin	442665	0	1	0	0	0	0
Liquorice	Uralenol	5315126	0	1	0	0	1	1
Liquorice	Uralenol-3-methylether	5315127	0	1	0	0	1	0
Liquorice	Sigmoidin-B	73205	0	1	0	0	1	1
Liquorice	uralenneoside	132594	1	1	0	0	1	1
Liquorice	schaftoside	442658	0	1	0	0	1	0
Liquorice	Nortangeretin	96506	1	1	0	1	1	1
Liquorice	neoisoliquiritin	5320092	0	1	0	0	0	0
Liquorice	Cyclobutanol,	145025	0	0	1	0	1	0

Liquorice	1-ethyl-Isoviolanthin	101422758	0	1	0	0	0	0
Liquorice	Isoglycyrol	124050	0	1	0	0	1	0
Liquorice	Isolicoflavonol	5318585	0	1	0	0	1	1
Liquorice	isoglycycoumarin	14187587	0	1	0	0	1	0
Liquorice	Isoliquiritin	5318591	0	1	0	0	0	0
Liquorice	Isoschaftoside	3084995	0	1	0	0	1	0
Liquorice	3,4,3',4'-Tetrahydroxy-2-methoxychalcone	6478421	1	1	0	0	1	1
Liquorice	Daidzein dimethyl ether	136419	1	1	0	0	1	0
Liquorice	4,2',4',alpha-Tetrahydroxydihydrochalcone	14632193	1	1	0	0	1	1
Liquorice	7-Acetoxy-2-methylisoflavone	268208	0	0	0	0	1	0
Liquorice	Artonin E	5481962	0	1	0	0	1	0
Liquorice	Vestitol	92503	0	1	0	0	1	1
Liquorice	Gancaonin G	480780	0	1	0	0	1	0
Liquorice	Gancaonin H	5481949	0	0	0	0	1	0
Liquorice	Gancaonin I	480777	0	1	0	0	1	0
Liquorice	Glyasperin A	5481963	0	1	0	0	0	0
Liquorice	Glycyrrhiza flavonol A	5317765	0	1	0	0	1	1
Liquorice	Corylifolinin	5281255	0	1	0	0	1	1
Liquorice	Kanzonol E	15516846	0	0	0	0	1	0
Liquorice	Licoagroisoflavone	636883	0	1	0	0	1	0
Liquorice	Odoratin	13965473	1	1	0	0	1	0
Liquorice	Phaseol	44257530	1	1	0	0	1	0
Liquorice	Astragalin	5282102	0	1	0	0	1	0
Liquorice	quercetin	5280343	1	1	0	0	1	1
Cistanche	(+)-Ledol	11074994	0	1	1	0	0	0
Cistanche	Leonurine	161464	1	0	0	0	0	0
Cistanche	Geniposidic acid	443354	0	1	0	0	1	0
Cistanche	Dauricine (8CI)	73400	0	0	0	0	0	0
Cistanche	Pulegone	442495	0	0	1	0	0	0

Cistanche	MTL	6251	0	1	0	0	0	0
Cistanche	8-epi-Loganic acid	158144	0	1	0	0	1	0
Cistanche	salidroside	159278	0	1	0	0	1	0
Cistanche	genistein	5280961	1	1	0	0	1	1
Cistanche	MENTHOL	1254	0	0	1	0	0	0
Cistanche	Propyl methyl trisulfide	5319765	0	0	0	0	1	0
Cistanche	cyanidol	128861	1	1	0	1	1	1
Cistanche	quercetin	5280343	1	1	0	0	1	1
Cistanche	Cistanin	44205314	0	1	1	0	1	0
Cistanche	Cistanoside F	101688189	0	1	0	0	0	0
Cistanche	Ethyl disulfide	8077	0	0	0	0	1	0
Cistanche	n-Decyl glucoside	62142	0	1	0	0	0	0
Cistanche	3,4,5,5-Tetramethyl-2-cyclopenten-1-one	579680	0	0	1	0	0	0
Mulberry	IEA	10685	0	1	1	0	1	0
Mulberry	WLN: T6NJ BG CQ	81136	0	1	0	0	0	0
Mulberry	Metacyl	12283	0	1	0	0	1	0
Mulberry	dIDP	135398667	0	1	0	0	1	0
Mulberry	1,8-cineole	2758	0	0	1	0	0	0
Mulberry	cis-resveratrol	1548910	0	1	0	0	0	1
Mulberry	geraniol	637566	0	0	0	0	0	0
Mulberry	(-)-alpha-Pinene	440968	0	0	1	0	0	0
Mulberry	CAM	5959	1	1	0	0	1	0
Mulberry	citric acid	311	0	0	0	0	1	0
Mulberry	MLT	4004	0	1	0	0	0	0
Mulberry	cyanidol	128861	1	1	0	1	1	1
Mulberry	myricetin	5281672	1	1	0	1	1	1
Mulberry	NOJ	29435	0	1	0	0	1	0
Mulberry	morin	5281670	1	1	0	0	1	1
Mulberry	quercetin	5280343	1	1	0	0	1	1
Hippophae Rhamnoides	pelargonidin	440832	0	1	0	0	1	1
Hippophae Rhamnoides	WLN: L6V BUTJ	13594	0	1	1	0	0	0
Hippophae	MBD	99555	1	1	0	1	1	0

Rhamnoides								
Hippophae Rhamnoides	3-O-Ethylascorbic acid	150736	0	1	0	0	0	0
Hippophae Rhamnoides	Bis-DHA	4613280	0	0	0	0	1	0
Hippophae Rhamnoides	(1R,2R,5S)-2-Isopropyl-5-methylcyclohexanol	6566020	0	0	1	0	0	0
Hippophae Rhamnoides	(2R,4R)-2-phenylchroman-4-ol	6923719	0	0	1	0	0	0
Hippophae Rhamnoides	(2S)-Flavanone	439652	0	0	1	0	1	0
Hippophae Rhamnoides	flavone	10680	0	1	0	0	1	0
Hippophae Rhamnoides	Flavonol	11349	0	1	0	0	1	0
Hippophae Rhamnoides	gamma-carotene	5280791	0	0	0	0	0	0
Hippophae Rhamnoides	glucofrangulin	99649	0	0	0	0	1	0
Hippophae Rhamnoides	isorhamnetin-3-O-galactoside	86328677	0	1	0	0	1	0
Hippophae Rhamnoides	Lichesterol	5281329	0	0	1	0	0	0
Hippophae Rhamnoides	propelargonidin	102115502	0	1	0	0	0	0
Hippophae Rhamnoides	(L)-alpha-Terpineol	443162	0	0	1	0	0	0
Hippophae Rhamnoides	citric acid	311	0	0	0	0	1	0
Hippophae Rhamnoides	MLT	4004	0	1	0	0	0	0
Hippophae Rhamnoides	24-epicampesterol	5283637	0	0	1	0	0	0
Hippophae Rhamnoides	Dodecanal	8194	0	0	0	0	0	0
Hippophae Rhamnoides	vitamin c	54670067	0	1	0	0	0	0
Hippophae Rhamnoides	24190-29-2	5498521	0	1	1	0	0	0
Hippophae	Furol	7362	0	1	0	0	0	0

Rhamnoides								
Hippophae Rhamnoides	cyanidol	128861	1	1	0	1	1	1
Hippophae Rhamnoides	myricetin	5281672	1	1	0	1	1	1
Hippophae Rhamnoides	Heriguard	1794427	0	1	0	0	1	0
Hippophae Rhamnoides	caffeic acid	689043	0	1	0	0	0	0
Hippophae Rhamnoides	rhein	10168	1	1	0	0	1	1
Hippophae Rhamnoides	Serotonin	5202	0	1	1	0	0	0
Hippophae Rhamnoides	cyclofenchene	79022	0	0	1	0	0	0
Hippophae Rhamnoides	Astragalin	5282102	0	1	0	0	1	0
Hippophae Rhamnoides	beta-Ionone	638014	0	0	1	0	0	0
Hippophae Rhamnoides	isorhamnetin	5281654	1	1	0	0	1	1
Hippophae Rhamnoides	D-Galacturonic acid, homopolymer	84740	0	1	0	0	0	0
Hippophae Rhamnoides	kaempferol	5280863	1	1	0	0	1	1
Hippophae Rhamnoides	Stigmasterol	5280794	0	0	1	0	0	0
Hippophae Rhamnoides	(+)-catechin	9064	1	1	1	0	1	1
Hippophae Rhamnoides	Isohexane	7892	0	0	0	0	0	0
Hippophae Rhamnoides	hesperidin	10621	0	0	0	0	0	0
Hippophae Rhamnoides	5,7-dihydroxy-2-(3-hydroxy-4-methoxyphenyl)chroman-4-one	676152	1	1	0	0	1	1
Hippophae Rhamnoides	Methose	5984	0	1	0	0	0	0
Hippophae Rhamnoides	procyanidin	107876	0	1	0	0	0	0
Hippophae Rhamnoides	Thiamine	1130	0	1	0	0	1	0

Hippophae Rhamnoides	progesterone	5994	1	0	1	0	1	1
Hippophae Rhamnoides	anthocyanin	145858	0	0	0	0	1	0
Hippophae Rhamnoides	l-Menthone	26447	0	1	1	0	0	0
Hippophae Rhamnoides	ent-Epicatechin	182232	1	1	1	0	1	1
Hippophae Rhamnoides	XYS	6027	0	1	1	0	0	0
Hippophae Rhamnoides	GLO	107526	0	1	1	0	0	0
Hippophae Rhamnoides	sucrose	5988	0	1	0	0	1	0
Hippophae Rhamnoides	Acetylfuran	14505	0	1	0	0	0	0
Hippophae Rhamnoides	quercetin	5280343	1	1	0	0	1	1

Note: RF, Random Forests; ANN, Artificial Neural Networks; SVM, Support Vector Machine.

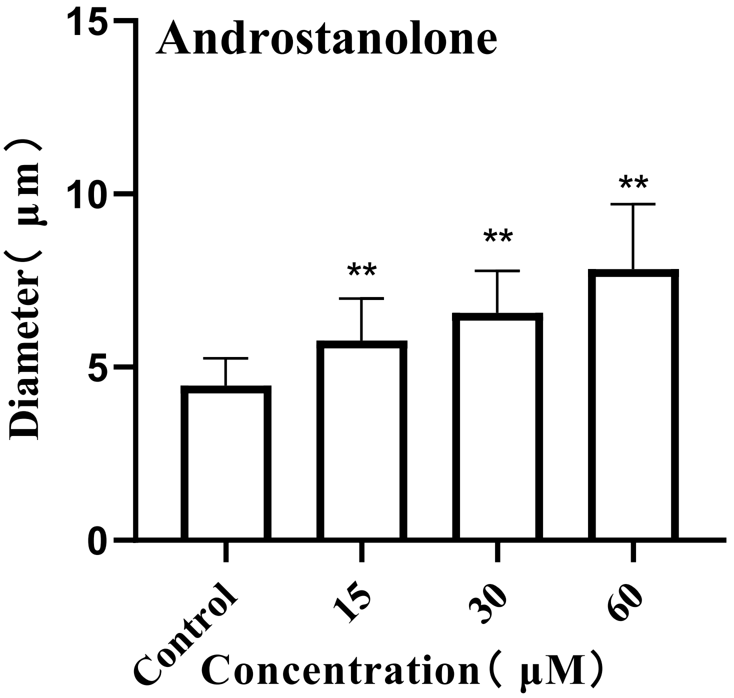

Fig. S4. Effects of androstanoloneon on the diameter of C2C12 myotubes. *P<0.05, **P<0.01 compared with control.

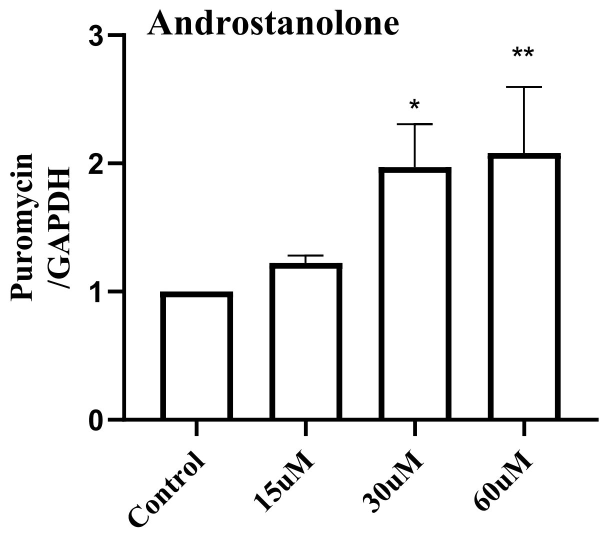

Figure S5. Effects of androstanoloneon on protein synthesis of C2C12 myotubes. $*P<0.05$, $**P<0.01$ compared with control.

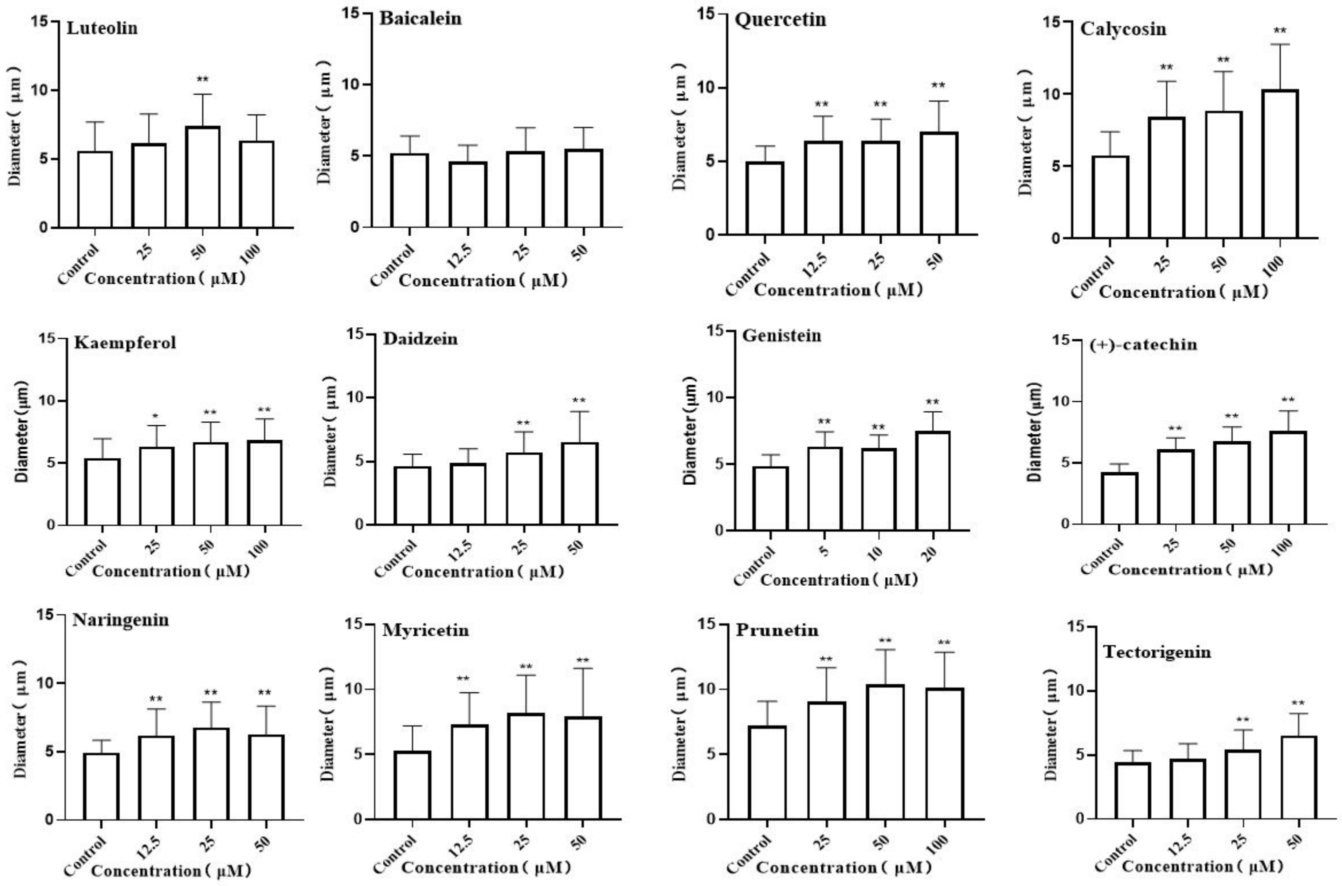

Figure S6. Effects of active components on the diameter of C2C12 myotubes. *, *P*<0.05; **, *P*<0.01.

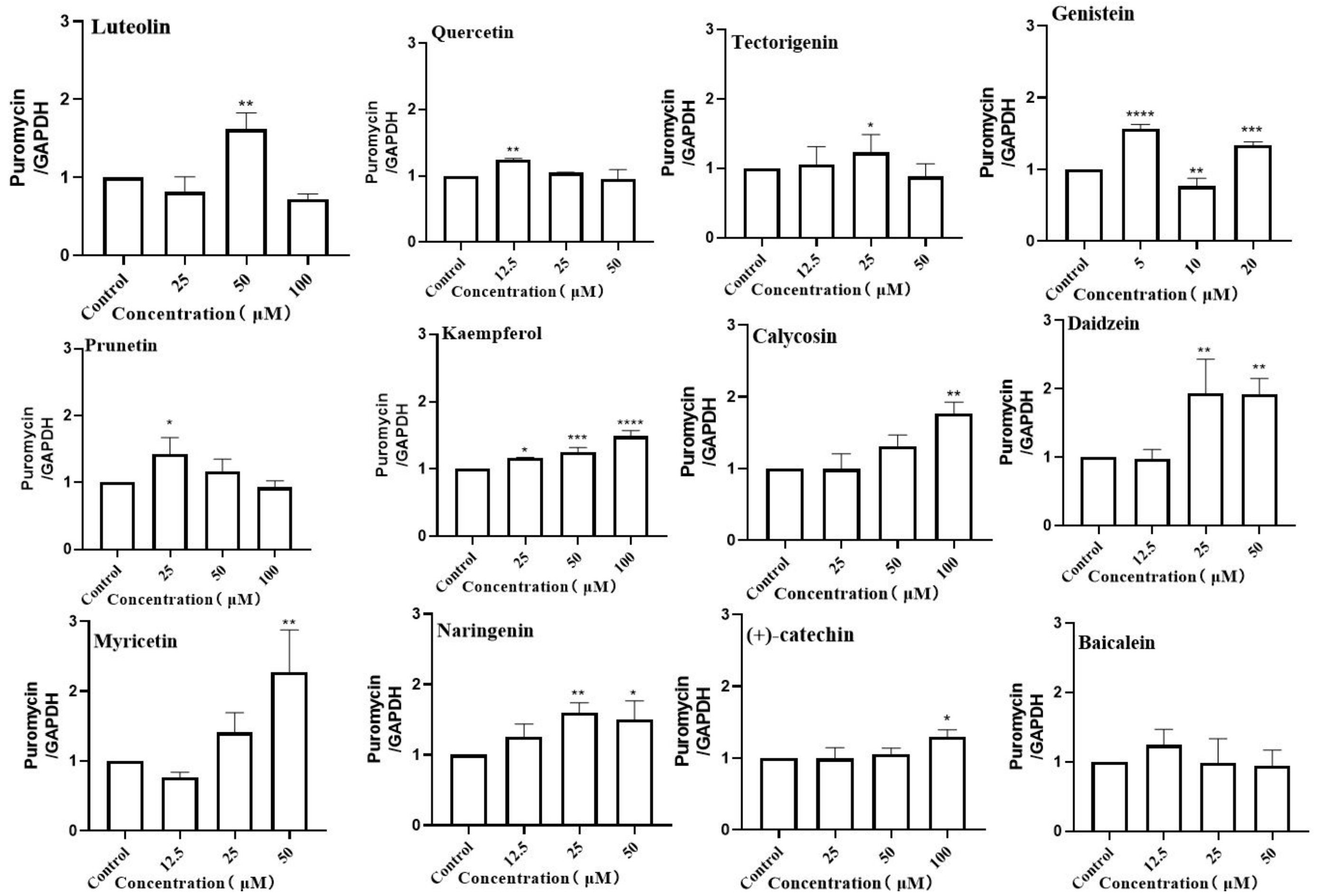

Figure S7. Effects of active components on the protein synthesis of C2C12. *, P<0.05; **, P<0.01.
